# Molecular networking-based GC/MS profiling of *Citrus japonica* Thunb. peel and pulp lipophilic fractions and their antimicrobial potential against diabetic foot ulcer pathogens

**DOI:** 10.1038/s41598-026-55298-y

**Published:** 2026-06-04

**Authors:** Nermin A. Ragab, Mona M. Marzouk, Hossam M. El-Masry, Salwa A. Kawashty, Reda S. Mohammed

**Affiliations:** 1 Pharmacognosy Department, Pharmaceutical and Drug Industries Research Institute, National Research Centre, 33 El Bohouth St, P.O. 12622, Cairo, 60014618 Egypt; 2 Phytochemistry and Plant Systematics Department, Pharmaceutical and Drug Industries Research Institute, National Research Centre, 33 El Bohouth St, P.O. 12622, Cairo, 60014618 Egypt; 3 Chemistry of Natural and Microbial Products Department, Pharmaceutical and Drug Industries Research Institute, National Research Centre, 33 El Bohouth St, P.O. 12622, Cairo, 60014618 Egypt

**Keywords:** Chemometrics, Diabetic foot infections, Fatty acid esters, *Citrus japonica*, Unsaponifiable matters, Biochemistry, Biotechnology, Microbiology

## Abstract

Diabetic ulcers are a serious complication of diabetes, often exacerbated by microbial infections that hinder wound healing. This study investigates the antimicrobial potential of non-polar constituents derived from the peel and pulp of *Citrus japonica* (kumquat) as natural antimicrobial agents for managing microbial infections associated with diabetic ulcers. Gas Chromatography-Mass Spectrometry (GC-MS)-based molecular networking enriches the annotation of 113 and 92 compounds in the peel and pulp lipophilic fractions, respectively. Moreover, preliminary in vitro antimicrobial assays demonstrated that the pulp non-polar extract possesses weak to moderate efficacy against common diabetic ulcer pathogens, including *Pseudomonas aeruginosa*,* Staphylococcus aureus*, and *Candida albicans*, with inhibition zone diameters (mm) of 13.67 ± 0.89, 19.33 ± 0.22 and 20.5 ± 0.17 compared to those of the standard 12.33 ± 0.22, 19.83 ± 0.39 and 10.71 ± 0.05 as well as MIC values (mg/mL) of 3.75 ± 0.04, 0.938 ± 0.002 and 1.88 ± 0.001, respectively. Furthermore, Principal Component Analysis (PCA)-based GC-MS revealed notable variations in the chemical profiles of the peel and pulp lipophilic fractions, with the pulp showing higher concentrations of trimethyl citrate and azelaic acid ester, compounds related to antibacterial and antifungal properties. This finding suggests that non-polar matter, especially from the pulp, could be a promising natural antimicrobial agent for treating diabetic ulcers. Further research is required to assess its efficacy in clinical applications and to explore its mechanisms of action in promoting wound healing and preventing infections.

**Supplementary Information:**

The online version contains supplementary material available at 10.1038/s41598-026-55298-y.

## Introduction

Diabetic foot ulcers are a serious consequence of poorly controlled diabetes. Globally, 537 million people are estimated to have diabetes, and 19% to 34% are expected to develop a diabetic foot ulcer at some point in their lifetime^1^. Previous studies proved that citrus essential oils are effective potential therapies for diabetic complications, including diabetic ulcers, through their antimicrobial activity against pathogenic bacteria and fungi, which are often involved in diabetic ulcer infections^2,3^. Moreover, citrus extracts and essential oils mitigate diabetic ulcers through their wound healing, antioxidant^4^, and anti-inflammatory effects^5^. *Kumquats* are tiny oval-shaped fruits, belonging to the genus *Citrus* of the family Rutaceae^5^. They are cultivated in four primary species: *Citrus japonica* Thunb., *C. crassifolia* Swingle, *C. margarita* (Lour.) Swingle, and *C. hindsii* (Champ. ex Benth.) Govaerts^6^. *C. japonica* (syn. *Fortunella japonica* Thunb.) is indigenous to South Asia and Asia-Pacific territories^7^ and is becoming more widely grown in South Africa due to its compatibility with subtropical climates. Its cultivation may increase in importance as market demands evolve and local populations seek diverse and healthy food options^8,9^.

In folk medicine, *C. japonica* has been used to treat coughs, colds, and other respiratory tract inflammation^5^. Unlike the other *Citrus* species, the kumquat fruit is usually eaten whole (peel and pulp altogether), because the composition of its peel essential oil differs from that of other citrus members^10^. Despite the various health benefits associated with *C. japonica* in Asian folk medicine, the phytochemical and biological properties remain notably understudied^11^.

The lipophilic constituents of *C. japonica* exhibit a wide range of functional properties, including aromatic attributes, insect-repellent, and antioxidant effects^6^ Previous studies have suggested that essential oils of *C. japonica* peel possess potent antimicrobial properties at microgram concentrations^6,12^ and shows an inhibitory effect on the proliferation of human prostate cancer cells by stimulating apoptosis and inflammation inhibition^13^. Furthermore, *C. japonica* oils possess effective antibacterial effects against *Escherichia coli*,* Bacillus strains*,* Salmonella typhimurium*,* S. aureus*, and *Lactobacillus bulgaricus*, in addition to antifungal effects against *C.* albicans^6^. From a nutritional perspective, essential oils add an incremental value to *C. japonica* fruit, as they influence flavour attributes and play a crucial role in maintaining human health^14^.

However, comparative studies on the lipophilic composition and biological activities of *C. japonica* peel and pulp are scarce. The incomplete understanding of the lipophilic compositions of *C. japonica* fruit and the chemical variability between its tissues (peels and pulp) may stem from limited research attention and the use of inadequate techniques for lipophilic identification in existing studies. Recently, GC-MS has been widely used to investigate bioactive natural compounds due to its sensitivity and effectiveness in quantitative and qualitative analysis^15^. Additionally, although research specifically on *C. japonica* lipophilic matter and diabetic ulcers is limited, the antimicrobial and wound-healing properties of citrus-derived compounds such as fatty acids, essential oils, and flavonoids are well established. Further research should focus on isolating and evaluating the lipophilic fractions of *C. japonica* peel and pulp for the treatment of diabetic ulcers. While GC-MS profiles of various *Citrus* species exist, this is the first study to compare peel vs. pulp lipophilic metabolomes of *C. japonica* using GNPS-based molecular networking. Furthermore, this study was designed as a preliminary in vitro screening of these fractions against diabetic-ulcer-relevant pathogens (*P. aeruginosa*, *S. aureus* and *C. albicans*).

## Materials and methods

### Plant material

*C. japonica* fruits were purchased from the Agriculture Research Centre (fruit section) (GPS: 30.02189° N, 31.21083° E) and were authenticated by the Fruit Department, Agricultural Research Institute, Fig. S1, National Research Centre (NRC). The peel and pulp were pressed until dry to prepare their voucher specimens (M301 and M302, respectively). They were deposited by Dr Mona M. Marzouk (Co-author) in the CAIRC (the herbarium of NRC).

### Preparation of extracts

Individually, the peel and pulp nonpolar fractions of *C. japonica* fruits were extracted at room temperature with ethanol/H_2_O (70:30) till exhaustion (4 L × 3 times). Under reduced pressure at 50 °C, the combined extracts evaporated using a rotary evaporator (Heidolph, Germany), yielding the hydroethanol extract of peel and pulp. In a separating funnel, about 10 g of each extract was dissolved in distilled water and repeatedly fractionated with dichloromethane till exhaustion. The collective fractions were filtered and evaporated to yield the dichloromethane fraction of each fruit part: non-polar fraction of peel (NPFPl, 1.5 g) and non-polar fraction of pulp (NPFPp, 0.78 g). Part of the NPFPl and NPFPp (0.5 g) was subjected to GC/MS analysis of the saponifiable and non-saponifiable matter to identify their chemical composition. The other part was biologically screened for its antimicrobial activities against selected microbial strains. All solvents used are of analytical grade ADWIC (Egypt).

### Chemical investigation of non-polar fractions

#### Preparation of saponifiable and unsaponifiable fractions from NPFPl & NPFPp

NPFpl or NPFPp (0.5 gm, each) was refluxed with 0.5 N alcoholic KOH (100 mL, 6 h, ) in a boiling water bath. The procedure was conducted as reported by^16^ to obtain unsaponifiable fractions (USFPl & USFPp) and fatty acid residues.

#### Preparation of fatty acid methyl esters

The residues of fatty acids that remained after the saponification step were dissolved separately in methanol (100 mL), mixed with sulphuric acid (0.5 mL), refluxed in a water bath (3 h, 100° C), then cooled, evaporated to dryness. The procedure was completed as reported by^16^ to obtain fatty acid ester fractions (FAEPl & FAEPp).

#### GC-MS analysis

USFPl, USFPp, FAEPl, and FAEPp were subjected separately to GC/MS analysis; GC/MS system (Agilent Technologies) was equipped with gas chromatograph (7890B) and mass spectrometer detector (5977 A) at Central Laboratories Network, National Research Centre, Cairo, Egypt. GC/MS analysis was equipped through the following conditions: fused silica GC capillary column (30 m length, 0.25 μm thickness, and 0.32 mm ID). 5% phenylpolysil phenylene siloxane (TR-5MS) was used as the stationary phase. the carrier gas is Helium (1mL/min, 13 psi). The temperature program is designed to be 60° C isothermal for 3 min, increased to 60–280 °C at a rate of 5° C per minute, and then to 260° C isothermal for 10 min. A mass spectrometer is employed as the detector with an electron ionization (EI) voltage (70 eV) and ion source temperature (200° C)^17^. Before each sequence, the column was baked at 280 °C for 60 min, and a procedural blank was run. The system was considered ready only when the blank showed a flat baseline (S/*N* < 3). The constituents were identified by matching their mass fragmentation spectra and retention times analysed by Gas/ Mass tools with those of the mass spectral libraries: NIST (Nat. Inst. St. Technol., USA), Wiley (Int., USA), and lipid library (https://lipidlibrary.aocs.org) as well as of the published data described by Adams 2019^18^ with similarity ≥ 80%.

#### GC/MS Molecular Networking

All raw GC-MS data files were converted to the universal .mzML open format using the MSConvert tool (Proteowizard Software, Version 3.0.19330, USA) with centroiding enabled. The converted files were then uploaded to the GNPS platform for processing with MS-Hub. Deconvolution was performed using the MS-Hub machine learning algorithm with the time unit set to minutes. All parameters related to peak symmetry and baseline adjustment were automatically determined internally by MS-Hub. After deconvolution, a spectral library search and molecular networking job was launched directly from the MS-Hub result page. The network was generated using the following GNPS parameters: fragment ion mass tolerance 0.5 Da, minimum matched fragment ions 6, score threshold 0.5, and minimum pairs cosine similarity 0.7. Spectral matching was performed against the GNPS spectral library (version 2023-11-01). Nodes with fewer than three MS/MS spectra or a cosine similarity below 0.7 were filtered out from the final network^19^. Two molecular networks (MNs) were established for GC/MS data of the unsaponifiable matters and the fatty acid methyl esters of the lipophilic fractions of *C. japonica* peel and pulp. Molecular networking enriches the annotation by leveraging information from interconnected nodes rather than individual components^20^. Visualization of the spectral network was conducted using Cytoscape 3.9.1.

#### Principal component analysis (PCA)

PCA was applied on the feature table obtained from GC- MSHUB, PCA biplot were constructed through JMP^®^, JMP Statistical Discovery LLC, Cary, NC”. A biplot combines loading plots and score plots, as the large databases generated from analytical results require chemometric evaluation. PCA was applied particularly to obtain low-dimensional representations of the data, and the PCA score plots were used to interpret different tissues of *C. japonica* fruits^21^.

### Antimicrobial investigation of non-polar fractions

#### Media and microbial strains

Nutrient agar medium (NA), which comprises (gm/L) yeast extract (2.0), peptone (5.0), meat extract (1.0), NaCl (5.0), and agar (15.0) with final pH value 7.4 ± 0.2, nutrient broth medium (NB) that has the following components (gm/L): yeast extract (2.0), peptone (5.0), meat extract (1.0), and NaCl (5.0), and final pH value 7.4 ± 0.2 for pathogenic bacterial strains, while the Sabouraud dextrose agar medium which comprises (gm/L) Dextrose (40.0), Peptic Digest of Animal Tissue (5.0), Pancreatic Digest of Casein (5.0), and agar (15.0) with final pH value 5.6 ± 0.2 at 25°C was used for *C. albicans* cultivation, All media components were purchased from Neogen Culture Media Formerly known as Lab M. Heywood, the Metropolitan Borough of Rochdale, Greater Manchester, England”. Three standard pathogenic strains were tested in the current study: one Gram-negative bacterium (*P. aeruginosa;* ATCC 27853), one Gram-positive bacterium (*S. aureus*; ATCC 6538), and one pathogenic fungal yeast (*C. albicans*; ATCC 10231), All microbial strains were obtained from the Bio-Evaluation Unit for Natural and Microbial Products, Laboratory no. 174 of the National Research Centre (NRC) through purchase from the American Type Culture Collection (ATCC, Manassas, Virginia, USA).

#### Procedure

A stock solution and the tested samples (NPFPl and NPFPp) were prepared by dissolving them separately in DMSO to give a concentration of 50.0 mg/mL. The qualitative antimicrobial evaluations of NPFPl and NPFPp took place using the NB medium according to Hafez et al.^22^. The inocula of pathogenic microbial strains were prepared from fresh overnight broth cultures using NB medium and incubated at 37 °C^23^. The sizes of inoculum of the tested pathogenic strains were prepared and adjusted to a 0.5 McFarland standard (1.5 × 10^8 CFU /mL)^24,25^. 20.0 µL inoculum size of each microorganism strain was separately inoculated into each plate containing 25.0 mL of the sterile nutrient agar medium. After the media cooled and solidified, the two tested samples were applied to a 0.6 cm well of the inoculated agar plates, which were prepared previously using a 0.6 cm cork borer and the well-diffusion method. Each well was separately filled with 70.0 µl of each sample. Triplicate trials of these samples were used^26^.

The previously prepared plates were placed for one hour in the refrigerator for more diffusion of these samples, then the plates were incubated at 37 °C for 24 h, and then the inhibition zones (IZ) that appeared were measured in mm^27^. Negative control: 5% DMSO, Gentamicin 10 mcg; standard antibiotic disc, Fluconazole (50 µg/disc) a standard antifungal disc. The experiment was performed three times independently (biological triplicates), and each independent experiment included triplicate wells for each sample (technical triplicates).

The samples that gave inhibition zones were passed to another antimicrobial test for the determination of minimum inhibitory concentration value (MIC) using Nutrient Broth medium, applying the microdilution broth method^26,28^.

The micro dilution broth method, take place using micro titer eliza plate, In this method each well of the Eliza plate was filled with 180.0 µL of sterile nutrient broth medium, then by serial dilution technique, each sample was used separately in first well by using the starting volume 100.0 µL that contains 50.0 mg/200 µL, of the used sample as a starting concentration, and by transfer of 100.0 µL from the first well to second well then transfer 100.0 µL from the second to the third, and so on to reach the final well that has sample concentration 0.78 mg of the used sample (6 dilutions were taken place), Then the wells were inoculated with the used tested pathogenic microbial strains separately by transfer 20.0 µL [a 0.5 McFarland standard (1.5 × 10^8 CFU /mL)] of these tested strains, finally each well contain final volume 200 µL, the microtiter plate incubate for 24 h at 37 °C, after that, the MIC was measured by using eliza reader throughout the absorption reading of this inoculated micro titer plate, where the mixture of this plate was shaken vigorously for 20 s and allowed to stand, the absorbance was then measured at 600.0 nm using a microplate multi-well reader (BioTek ELx800 Absorbance Microplate ELISA Reader, Agilent company, USA). The lower absorbance of the reaction mixture, which is equal to the absorption of the blank sample, is referred to as MIC activity^26,28^.

### Statistical analysis

Statistical analyses were performed using SPSS (Statistical Package for the Social Sciences) followed by Duncan’s multiple range test was employed to evaluate the differences among groups, and results were considered statistically significant at *P* ≤ 0.05. In ANOVA assumptions; Normality was checked by Shapiro Wilk test (*p* > 0.05) and homogeneity of variances by Levene’s test (*p* > 0.05). Both assumptions were met for all datasets.

Duncan’s multiple range test was used for post hoc comparisons because it is appropriate for pairwise comparisons of a single factor (sample type) with three or more groups; we chose Duncan’s as it is more sensitive for detecting differences among close means in agricultural/biological studies. The same threshold (*p* ≤ 0.05) was applied.

## Results

### Chemical investigation of non-polar fractions and GC/MS-based molecular networking

**Fig. 1:**
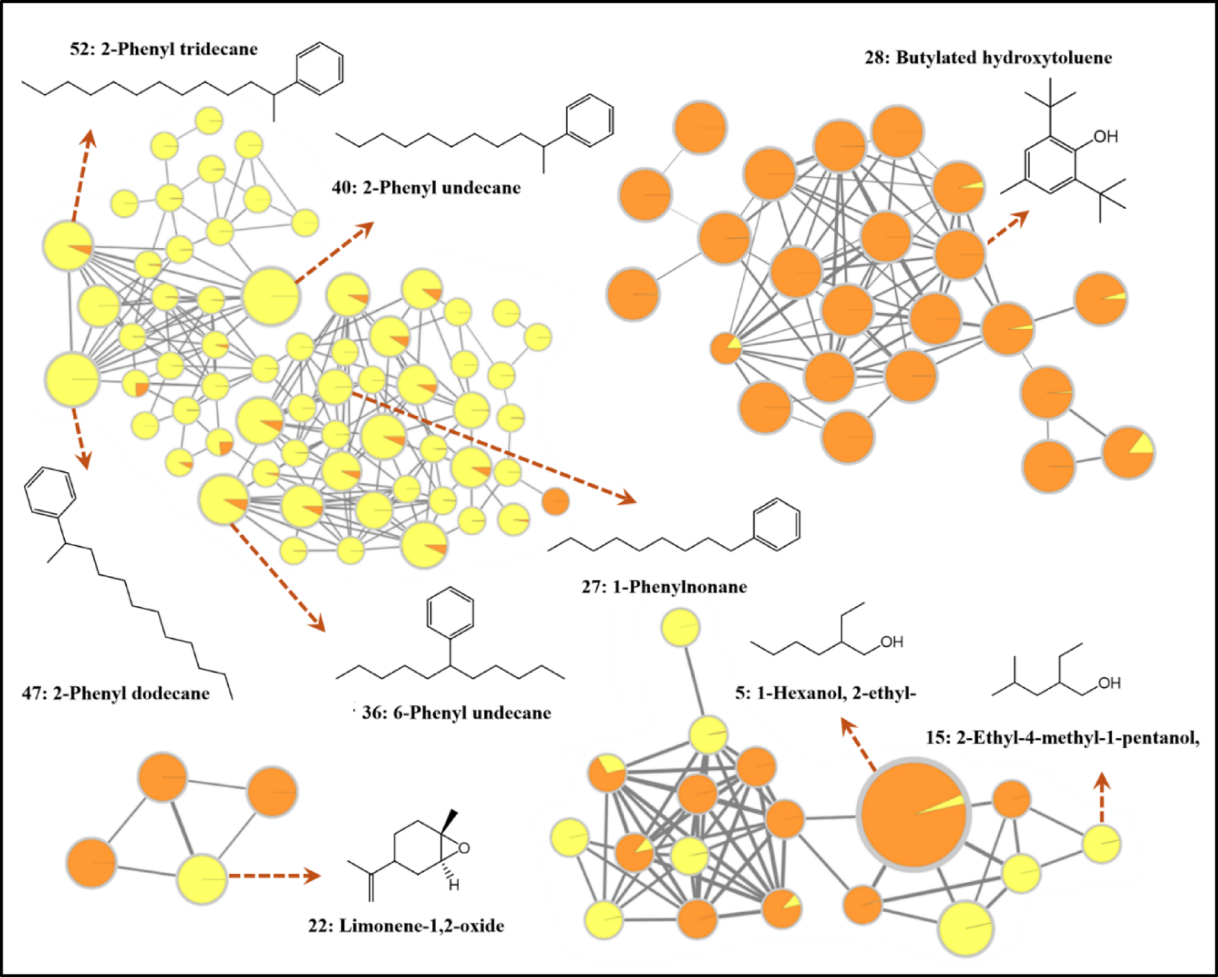
Groups of interest for the GC/MS Molecular network of unsaponified fractions of *Citrus japonica*. Node color: distribution of compound among peel and pulp (USFPl with orange and USFPp with yellow). Nodes represent MS/MS spectra. Node size: total sum of intensity of the corresponding ion.

**Fig. 2:**
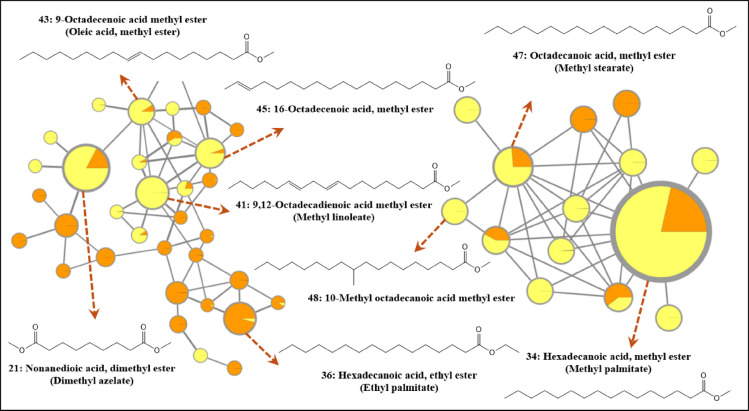
Groups of interest for the GC/MS Molecular network of fatty and carboxylic acids esters of *Citrus japonica*. Node color: distribution of compound among fatty acid esters of peels and pulp (FAEPl with orange and FAEPp with yellow). Nodes represent MS/MS spectra. Node size: total sum of intensity of the corresponding ion.

GC/MS analysis revealed a significant difference in the unsaponifiable matter (USFPl and USFPp) (Table 1) and fatty acid esters (FAEPl and FAEPp) of saponifiable matter (Table 2) among the studied peel and pulp extracts. Two GC/MS-based GNPS molecular networks were created. Only matches between investigated and library spectra with a score above 0.5 and at least 6 matched fragments were considered and confirmed through published data^18^. MN groups of interest are represented in Figs. 1 and 2, for the unsaponifiable and saponifiable matters, respectively.

The GC/MS investigation of the USFPl and USFPp (Fig. S2 & Fig. S3) revealed the identification of 49 and 46 compounds constituting total areas of 82.646 and 94.368, respectively (Table 1). The oxygenated compounds constituted 37. 28% and 28.647% while the non-oxygenated compounds constituted 44.038% and 62.581%, respectively. Phenyl hydrocarbons are major compounds in two fractions, constituting 33.07% and 60.63%, respectively. Among the identified unsaponifiable compounds, butylated hydroxytoluene (10.58%) and limonene (2.23%) were present in the peel fraction in significantly higher amounts than in the pulp (1.67% and 0.26%). In contrast, limonene oxide accumulated in the pulp (14.448%), while only a trace amount was detected in the peel (0.13%).

Similarly, FAEPl and FAEPp were analyzed by GC/MS (Fig. S4 & Fig. S5), which resulted in the identification of 53 and 46 compounds constituting total area % (71.27 and 82.96), respectively (Table 2). The percentages of saturated & unsaturated fatty acids were close in both parts, as (32.920 and 30.372) & (13.522 and 11.008), respectively, for (FAEPl and FAEPp). Most of the fatty acids identified belong to dicarboxylic structures. The major compounds in both parts were hexadecanoic acid methyl ester (methyl palmitate) with area % (9.597% and 22.872%), followed by nonanedioic acid dimethyl ester (dimethyl azelate) (6.518% and 7.51%), then octadecanoic acid, methyl ester (stearic acid) (2.193% and 4.015%), respectively. The pulp and peel were rich in carboxylic acids (mono-, di-, and tricarboxylic acid). The latter acid was detected in the pulp and identified as trimethyl citrate with a relative percentage of 7.3%.

**Table 1:** GC/MS analysis of unsaponified fractions of *Citrus japonica* (USFPl and USFPp).

No.	*R*_t_ (min)	Compounds	Area %		BP	MW	Mol. formula
			USFPl	USFPp			
1	7.34	3,3-Diethoxy-1-propanol	0.58	-	75	148	C_7_H_16_O_3_
2	8.45	Decane	0.002	0.34	57	142	C₁₀H₂₂
3	9.28	Limonene	2.23	0.26	68	136	C_10_H_16_
4	9.35	Allyl benzyl ether	0.12	2.4	91	148	C_10_H_12_O
5	9.47	1-Hexanol, 2-ethyl-	20.7	0.46	57	130	C_8_H_18_O
6	10.26	3,3-Dimethyl-Hexane	0.42	0.3	43/57	114	C_8_H_18_
7	10.68	Linalool oxide	-	0.17	43/94	170	C_10_H_18_O_2_
8	10.86	2-Nonanol	0.16	0.98	41	144	C_9_H_20_O
9	11.25	n-Undecane	0.09	0.11	71	156	C_11_H_24_
10	11.29	4-Methyl -octane	0.46	-	43	128	C_9_H_20_
11	11.53	1-Heptanol, 3-methyl	0.35	-	43	130	C_8_H_18_O
12	11.53	Pulegone	-	0.27	67/81	152	C_10_H_16_O
13	12.10	2,4-Dimethyl-Heptane	1.16	0.02	57	128	C_9_H_20_
14	12.35	2-Methylhept-6-en-3-one	0.16	3.76	55	126	C_8_H_14_O
15	12.86	2-Ethyl-4-methyl-1-pentanol	-	0.049	57/43	130	C_8_H_18_O
16	13.07	n-Dodecane	0.18	0.11	57	170	C_11_H_24_
17	13.26	3-Methy undecane	0.009	0.11	57	170	C_12_H_26_
18	13.31	2,2-diethoxy Ethanamine	0.35	1.04	47/103	133	C_6_H_15_NO_2_
19	14.09	2,4-Dimethyl-decane	0.31	1.94	57	170	C_12_H_26_
20	16.80	n-Tridecane	0.007	0.14	57	184	C_13_H_28_
21	18.11	8-*p*-Menthadien-1,2-diol	0.61	-	71	152	C_10_H_16_O
22	18.14	Limonene 1,2-oxide	0.13	14.448	43	152	C_10_H_16_O
23	18.19	1,8-p-Menthadien-7-ol (Perillyl alcohol)	3.03	0.21	79	152	C_10_H_16_O
24	18.75	2,2-Diethoxy-N, N-dimethyl-ethanamine	0.56	-	75/103	161	C_8_H_19_NO_2_
25	18.76	Methyl-2-phenyl hexane	-	0.25	105	176	C_13_H_20_
26	19.19	1-Tetradecene	1.08	0.18	55	196	C_14_H_28_
27	20.64	1-Phenylnonane	0.03	2.04	91	204	C_15_H_24_
28	22.29	Butylated hydroxytoluene	10.58	1.67	220	220	C_15_H_24_O
29	22.41	2,6-Bis(1-methylpropyl) Phenol	0.86	0.10	191	206	C_14_H_22_O
30	22.78	5-Phenyl decane	0.66	-	91	218	C_16_H_26_
31	23.00	4-Phenyl decane	0.56	2.10	91	218	C_16_H_26_
32	23.42	3- Phenyl decane	0.73	2.62	91	218	C_16_H_26_
33	22.37	Caryophyllene	0.72	0.001	79	204	C_15_H_24_
34	23.77	2-Phenyl decane	1.33	2.30	105	218	C_16_H_26_
35	23.97	1-Hexadecene	1.17	-	55	224	C_16_ H_32_
36	24.26	6-Phenyl undecane	0.86	3.79	161	232	C_17_H_28_
37	24.36	5-Phenyl undecane	1.87	0.14	91	232	C_17_H_28_
38	24.97	4-Phenyl undecane	1.80	2.87	91	232	C_17_H_28_
39	25.08	3-Phenyl undecane	2.10	4.66	91	232	C_17_H_28_
40	25.51	2-Phenyl undecane	1.43	4.18	105	232	C_17_H_28_
41	25.99	6-Phenyl dodecane	1.35	0.19	91	246	C_18_H_30_
42	27.11	5-Phenyl dodecane	3.29	0.10	91	246	C_18_H_30_
43	27.22	4-Butyl-indan-5-ol	-	3.87	147	190	C_13_H_18_O
44	28.37	5-Octadecene	1.08	-	55	252	C_18_H_36_
45	27.82	4-Phenyl dodecane	2.98	3.80	91	246	C_18_H_30_
46	27.92	3-Phenyl dodecane	4.58	4.58	91	246	C_18_H_30_
47	28.61	2-Phenyldodecane	2.65	7.67	105	246	C_18_H_30_
48	29.14	6-Phenyl tridecane	1.53	4.44	91	260	C_18_H_30_
49	29.28	5-Phenyl tridecane	1.13	3.05	91	260	C_19_H_32_
50	29.52	4-Phenyl tridecane	1.21	3.00	91	260	C_19_H_32_
51	29.98	3-Phenyl tridecane	1.15	3.79	91	260	C_19_H_32_
52	30.74	2-Phenyl tridecane	1.83	5.31	105	260	C_19_H_32_
53	32.17	3-Eicosene	1.58	-	55	280	C_20_H_40_
54	37.50	n-Tricosane	-	0.13	57	324	C_23_H_48_
55	40.64	n-Pentacosane	0.47	0.16	71	352	C_25_H_25_
Total identified compounds			82.646	94.368			
a. Oxygenated			37.28	28.647			
• Phenolic compounds			11.44	1.77			
• Oxygenated monoterpene			3.77	15.358			
• Alcohols			21.79	1.489			
• Ketones			0.16	3.76			
• Miscellaneous			0.12	6.27			
b. Non-oxygenated			44.038	62.581			
• Phenyl HC			33.07	60.63			
• Straight chain saturated HC			0.749	0.99			
• Branched saturated HC			2.359	2.62			
• Unsaturated HC			4.91	0.18			
• Non-oxygenated monoterpene			2.23	0.26			
• Sesquiterpenenoids			0.72	0.001			
c. Nitrogenous oxygenated compounds			0.91	1.04			
Total non-identified compounds			16.36	5.992			

BP; Base peak, Mw; Molecular weight, R_t_; retention time, HC; hydrocarbons, USFPl; unsaponifiable fraction of peel, USFPp; unsaponifiable fraction of pulp.

**Table 2:** GC/MS analysis of fatty and carboxylic acids esters of *Citrus japonica* (FAEPl and FAEPp).

No.	*R*_t_ (min)	Compounds	Area %		BP	MW	Mol. Formula
			FAEPl	FAEPp			
1	10.56	Methyl 2-furoate	-	0.844	95	126	C_6_H_6_O_3_
2	12.66	Butanedioic acid, dimethyl ester (Dimethylsuccinate)	-	1.024	115	146	C_6_H_10_O_4_
3	13.82	Butanedioic acid, methyl-, dimethyl ester (Dimethyl methyl succinate)	-	0.263	129/59	160	C_7_H_12_O_4_
4	14.75	Methyl benzoate	0.463	-	105	136	C_8_H_8_O_2_
5	15.16	Butanedioic acid, ethyl methyl ester (Methyl ethyl succinate)	0.483	-	-	160	C_7_H_12_O_4_
6	16.49	3-Methyl glutaric acid dimethyl ester	0.362	1.373	114/101	174	C_8_H_14_O_4_
7	17.22	Methyl − 10-Pentadecenoate	0.342	-	55	254	C_16_H_30_O_2_
8	17.33	Benzeneacetic acid, methyl ester	0.282	0.318	91	150	C_9_H_10_O_2_
9	17.43	Butanedioic acid, diethyl ester (Diethyl succinate)	0.563	-	101/129	174	C_8_H_14_O_4_
10	17.99	Propyl isobutyrate	0.101	1.205		130	C_7_H_14_O_2_
11	19.29	Hexanedioic acid, dimethyl ester (Dimethyl adipate)	0.161	0.097	114/101	174	C_8_H_14_O_4_
12	20.58	Methyl octanoate	0.553	0.083	74	158	C_9_H_18_O_2_
13	22.09	Heptanedioic acid, dimethyl ester (Dimethyl pimelate)	0.021	0.789	115/74	188	C_9_H_16_O_4_
14	23.15	2-Propenoic acid, 3-phenyl-, methyl ester (Methyl cinnamate)	1.267	0.003	131/103	162	C_10_H_10_O_2_
15	23.98	1-Ethyl heptanedioate (Pimelic acid monoethyl ester)	0.704	0.006	88/74	188	C_9_H_16_O_4_
16	23.98	Heptanedioic acid, dimethyl ester (Dimethyl pimelate)	0.362	-	125	188	C_9_H_16_O_4_
17	24.54	1, 2, 3-Prop-1-ene tricarboxylic acid, trimethyl ester	-	0.928	153	216	C_9_H_12_O_6_
18	24.81	Octanedioic acid, dimethyl ester	6.237	1.301	138	202	C_10_H_18_O_4_
19	25.36	Citric acid, trimethyl ester (Trimethyl citrate)	-	7.3	143/101	234	C_9_H_14_O_7_
20	25.74	Heptanedioic acid, diethyl ester	0.261	-	125	216	C_11_H_20_O_4_
21	27.23	Nonanedioic acid, dimethyl ester (Dimethyl azelate)	6.518	7.51	152	216	C_11_H_20_O_4_
22	28.12	9- Hexadecenoic acid methyl ester	-	0.346	55	268	C_17_H_32_O_2_
23	28.15	Octanedioic acid, diethyl ester (Diethyl suberate)	1.127	0.001	143	230	C_12_H_22_O_4_
24	28.34	Ethyl dodecanoate (Ethyl laurate)	0.744	0.166	88/74	228	C_14_H_28_O_2_
25	29.55	1,10-Decanedioic acid, dimethyl ester (Dimethyl sebacate)	0.785	2.907	125/74	230	C_12_H_22_O_4_
26	30.17	2-Propenoic acid, 3-(4-methoxyphenyl)-, methyl ester (Methyl-p-methoxycinnamate)	0.008	0.305	161/133	192	C_11_H_12_O_3_
27	31.67	1,11-Undecanedioic acid dimethyl ester	0.463	0.397	213/139	244	C_13_H_24_O_4_
28	31.87	3,4- dimethyl benzoic acid methyl ester	0.010	0.221	133	164	C_10_H_12_O_2_
29	32.56	Decanedioic acid diethyl ester (Diethyl sebacate)	0.342	-	97	258	C_14_H_26_O_4_
30	31.24	Tetradecanoic acid, methyl ester	1.247	-	74	242	C_15_H_30_O_2_
31	32.71	Tetradecanoic acid, ethyl ester	1.207	0.042	88	256	C_16_H_32_O_2_
32	33.36	Isopropyl tetradecanoic acid methyl ester (Tetradecanoic acid, 1-methyl ethyl ester)	0.301	-	74	270	C_17_H_34_O_2_
33	33.55	9-Hexadecenoic acid, methyl ester (methyl palmitoleate)	1.83	1.273	55	268	C_17_H_32_O_2_
34	35.42	Hexadecanoic acid, methyl ester (Methyl palmitate)	9.597	22.872	74	270	C_17_H_34_O_2_
35	35.52	13-Methyl pentadcanoic acid methyl ester	0.004	0.249	74/87	284	C_18_H_36_O_2_
36	36.71	Hexadecanoic acid, ethyl ester (Ethyl palmitate)	7.465	0.208	88	284	C_18_H_36_O_2_
37	36.81	14- Methyl heptadecanoic acid methyl ester	-	0.235	74/87	298	C_19_H_38_O_2_
38	37.60	Hexadecanoic acid 2-hydroxy-methyl ester (Hydroxypalmitate)	1.408	7.864	97/83	286	C_17_H_34_O_3_
39	38.55	Heptadecanoic acid, ethyl ester	0.664	-	88	298	C_19_H_38_O_2_
40	39.36	9,12,15 octadecatrienoic acid methyl ester	1.830	0.152	79	292	C_19_H_32_O
41	38.58	9,12-Octadecadienoic acid methyl ester (Methyl linoleate)	0.163	5.524	67/81	294	C_19_H_34_O_2_
42	38.74	6, 9- Octadecadienoic acid methyl ester	0.724	-	67	294	C_19_H_34_O_2_
43	38.78	9-Octadecenoic acid methyl ester (Oleic acid, methyl ester)	1.147	4.430	55/87	296	C_19_H_36_O_2_
44	38.80	9,12-Octadecadienoic acid ethyl ester (Ethyl linoleate)	1.227	0.001	67/88	308	C_20_H_36_O_2_
45	38.83	16-Octadecenoic acid, methyl ester	0.382	4.264	97/55	296	C_19_H_36_O_2_
46	39.08	9-Octadecenoic acid ethyl ester (Ethyl Oleate)	0.624	-	95	310	C_20_H_38_O_2_
47	39.14	Octadecanoic acid, methyl ester (Methyl stearate)	2.193	4.015	74	298	C_19_H_38_O_2_
48	40.29	10-Methyl octadecanoic acid methyl ester	-	0.387	74/87	312	C_20_H_40_O_2_
49	40.33	Octadecanoic acid, ethyl ester (Stearic acid, ethyl ester)	2.193	-	88	312	C_20_H_40_O_2_
50	42.54	9,10-dihydroxy octadecanoic acid, methyl ester	-	2.409	87	330	C_19_H_38_O_4_
51	43.04	14-Oxo-nonadec-10-enoic acid methyl ester	1.710	-	99	324	C_20_H_36_O_3_
52	43.62	5,8,11-Heptadecatriynoic acid, methyl ester	1.911	-	101	272	C_18_H_24_O_2_
53	45.78	Docosanoic acid, methyl ester	0.766	-	74	354	C_23_H_46_O_2_
54	45.80	Methyl-20-methyl-heneicosanoate	0.966	0.914	74/87	354	C_23_H_46_O_2_
55	46.76	Docosanoic acid, ethyl ester	0.362	-	88	368	C_24_H_48_O_2_
56	48.37	Tricosanoic acid, ethyl ester	2.052	0.941	74/87	382	C_25_H_50_O_2_
57	48.73	Tetracosanoic acid, methyl ester	0.905	-	74	382	C_25_H_50_O_2_
58	49.2	Methyl 2-hydroxydocosanoate	0.060	0.332	57/97	370	C_23_H_46_O_3_
59	50.61	Methyl 2-hydroxytetracosanoate	-	0.373	57/97	398	C_25_H_50_O_3_
60	51.49	Hexacosanoic acid, methyl ester	0.443	0.263	74	410	C_27_H_54_O_2_
61	52.42	Hexacosanoic acid ethyl ester	0.342	0.028	88	424	C_28_H_56_O_2_
62	54.79	Octacosanoic acid methyl ester	0.865	-	143	438	C_29_H_58_O_2_
63	56.06	Heptacosanoic acid ethyl ester	0.422	0.010	88	452	C_30_H_60_O_2_
Total identified compounds			71.27	82.96			
a. Fatty acid methyl esters							
• Saturated Fatty acids ester			32.920	30.372			
• Unsaturated fatty acids ester			13.522	11.008			
• Hydroxyl saturated fatty acids ester			1.469	10.979			
• Dicarboxylic fatty acid ester			18.108	13.008			
b. Carboxylic acid methyl esters							
• Mono carboxylic acid ester			2.492	3.270			
• Dicarboxylic acids ester			1.046	3.287			
• Tricarboxylic acid ester			-	11.034			
c. Miscellaneous							
• Oxo unsaturated fatty acid ester			1.710	-			
Total non-identified compounds			28.73	17.04			

BP; Base peak, Mw; Molecular weight, R_t_; retention time, FAEPl; fatty acid methyl esters of peel, FAEPp; fatty acid methyl esters of pulp.

### Principal component analysis

**Fig. 3:**
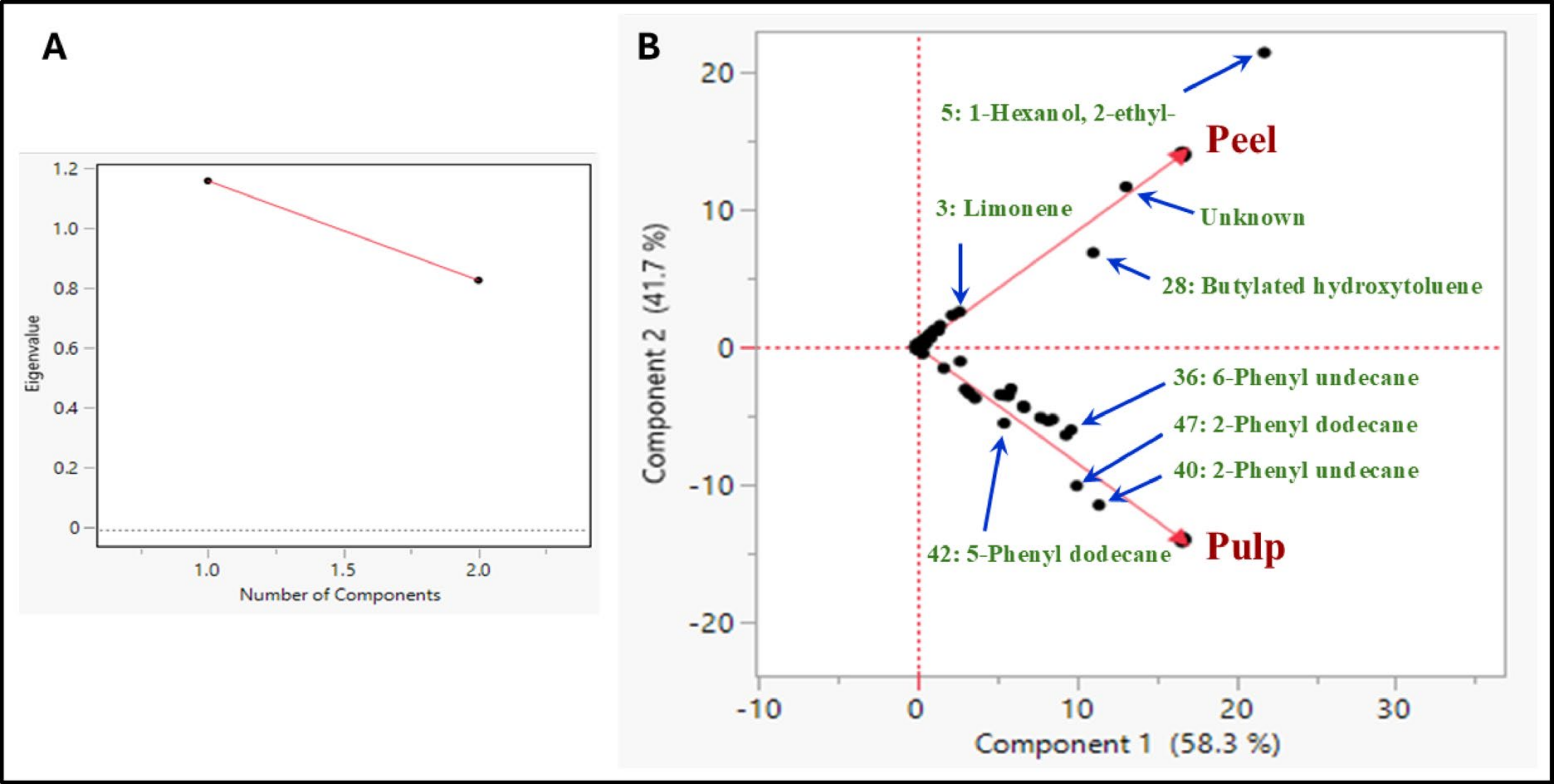
**A**: Scree plot of unsaponifiable matter, **B**: Principal component analysis (PCA) biplot (combination of variables on loadings plot and samples on scores plot) of unsaponifiable matter from gas chromatography-mass spectrometry of *Citrus japonica* peels and pulps.

**Fig. 4:**
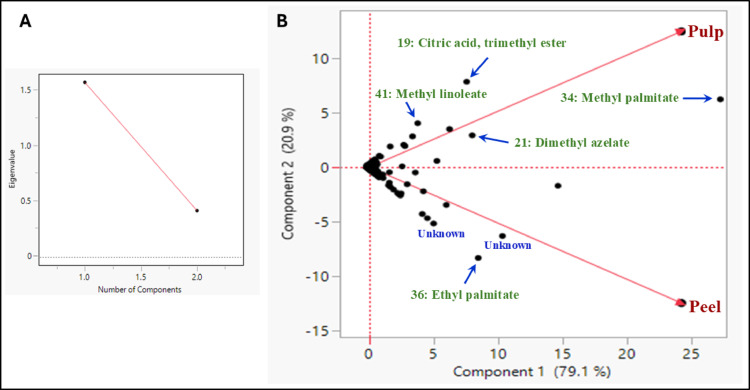
**A**: Scree plot of fatty acid methyl/ethyl esters, **B**: principal component analysis (PCA) biplot (combination of variables on loadings plot and samples on scores plot) of saponifiable matter from gas chromatography-mass spectrometry of *Citrus japonica* peels and pulps.

PCA is a data analysis method commonly used in chemometric metabolomic research. Dimensionality-reduction techniques such as PCA typically require investigators to determine the number of components to retain. Visual assessment of the scree plot is crucial for determining the proper number of components to keep. The scree plot is an experimental graphic method constructed of the eigenvalues (y-axis) against the components (x-axis), and it represents the maximum number of components to keep^29^(Figs. 3A and 4A). The PCA biplot for datasets acquired from both the saponifiable and unsaponifiable fractions showed a clear segregation between the peels and pulp of *C. japonica.* Since the biplot analysis of both the saponifiable and unsaponifiable fractions demonstrated adequate variance coverage—explaining 79.1% and 20.9% of the total variance along PC1 and PC2, respectively, for the saponifiable matter, and 58.3% and 41.7% for the unsaponifiable matter (Figs. 3A and 4A)^21^.

For the unsaponifiable and saponifiable matters, component 2 (PC2) revealed a clear segregation between the peel and pulp samples and showed a distinct metabolic profile unique to each tissue. Unlike component 2, peels and pulp are pulled together at the positive side of component 1 (PC1) (Figs. 3B and 4B).

In the unsaponifiable matter, the positive side of Component 2, associated with the peels, indicates an abundance of constituents such as 1-hexanol, 2-ethyl-, butylated hydroxytoluene, and limonene. Pulp appeared rich in alkylbenzene primary compounds, 2-phenyl undecane, 2-phenyldodecane, and 5-phenyl dodecane compared to peel (Fig. 3B). In the saponifiable matter, the positive loadings of the saponifiable constituents associated with pulp are methyl palmitate, trimethyl citrate, octadecadienoic acid methyl ester, and nonanedioic acid dimethyl ester (dimethyl azelate). Controversy, the negative loadings of the saponifiable constituents associated with the peel are hexadecanoic acid ethyl ester (ethyl palmitate) (Fig. 4B). The fruit’s pulp main role is nutrient storage and aiding the seed development. Methyl palmitate and citric acid ester may participate in energy storage and metabolic activities essential for these processes^30,31^. Whereas the peel serves as a shield against environmental threats such as pathogens, UV radiation, and mechanical injury, ethyl palmitate, a fatty acid ester rich in peels, may help reinforce the protective wax layers of the peel^32^.

The PCA finding highlights the different functional roles of peel and pulp, with the peel storing limonene as a protective mechanism and pulp accumulating aromatic alkylbenzenes for nutrient storage and seed protection, potentially drawing seed dispersers^33–35^. These metabolomic profile variations may be attributed to differences in multiple metabolic pathways establishment^36^.

### Antimicrobial investigation of non-polar fractions

**Fig. 5:**
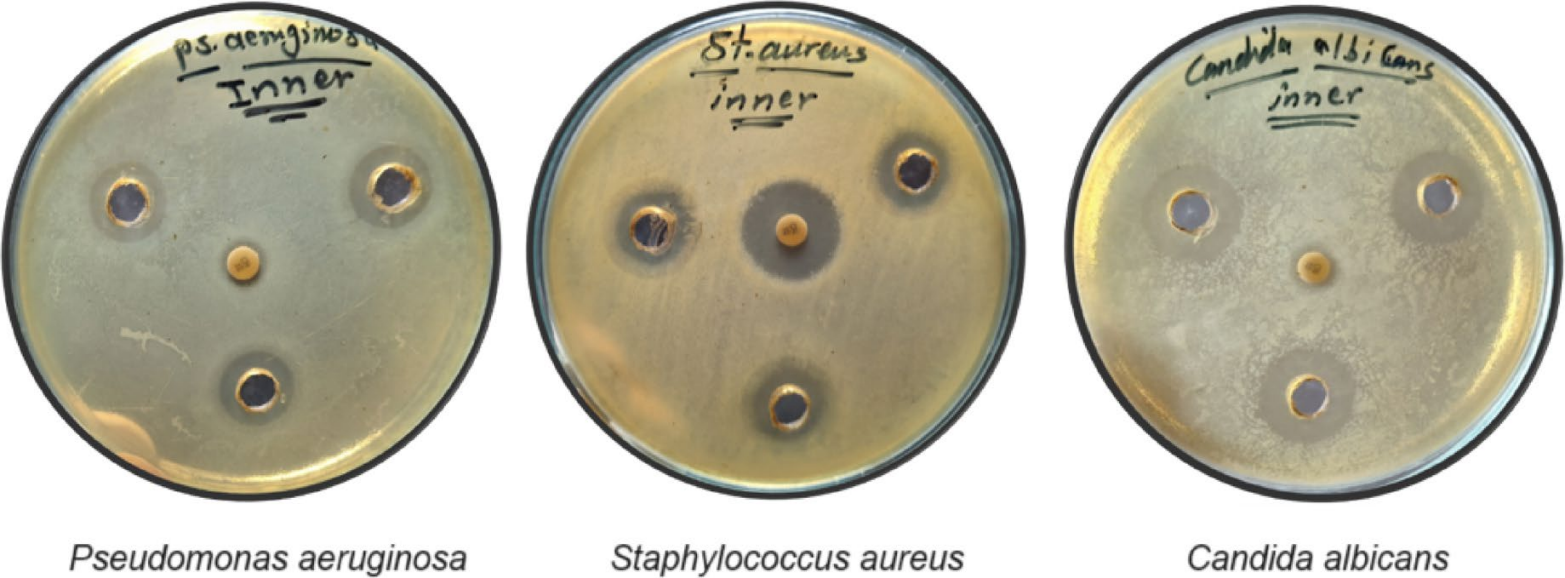
Inhibition zone diameter (mm) applying the well diffusion technique of NPFPp against the tested microbial strains.

The antimicrobial evaluation of NPFPl and NPFPp was screened against three selected microbial strains. The positive antimicrobial effects were observed for NPFPp against a Gram-negative bacterium (*P. aeruginosa*), a Gram-positive bacterium (*S. aureus*), and a fungal yeast strain (*C. albicans*) with inhibition zone diameters (mm) 13.67 ± 0.89, 19.33 ± 0.22, and 20.5 ± 0.17 compared to those of standard 12.33 ± 0.22, 19.83 ± 0.39, and 10.71 ± 0.05, respectively (Table 3; Fig. 5). One-way ANOVA followed by Duncan’s multiple range test (*p* ≤ 0.05) revealed that antimicrobial inhibition zone against *P. aeruginosa*: NPFPp (letter a) was significantly more active than gentamicin (letter b), whereas against *S. aureus*: NPFPp and gentamicin shared the same letter (a), indicating no significant difference. The results against *C. albicans*: NPFPp (letter a) were significantly more active than fluconazole (letter b).

The MIC values of NPFPp were 3.75 mg/mL for *P. aeruginosa*, 0.938 mg/mL for *S. aureus*, and 1.88 mg/mL for *C. albicans*, all significantly higher than those of the positive controls (different letters, *p* ≤ 0.05). No effects were observed for NPFPl.

**Table 3:** Inhibition zone diameter (mm) applying the well diffusion method and MIC (mg/mL) applying the microdilution broth method of NPFPp.

Tested organisms	Inhibition zone diameter (mm)		MIC (mg/mL)	
	NPFPp	CN	NPFPp	CN
*Pseudomonas aeruginosa*	13.67^a^±0.89	12.33^b^±0.22	3.75^a^±0.04	0.125^b^±0.0009
*Staphylococcus aureus*	19.33^a^±0.22	19.83^a^±0.39	0.938^a^±0.002	0.118^b^±0.0003
*Candida albicans*	20.5^a^ ± 0.17	10.71^b^±0.05	1.88^a^±0.001	0.06^b^±0.0001

Data are presented as mean ± SD. One-way ANOVA was used for data analysis (*n* = 3, *P* ≤ 0.05) the NPEpp; non-polar fraction of pulp, CN; Reference sample [(Gentamicin 10 mcg; standard antibiotic disc, Fluconazole (50 µg/disc) a standard antifungal disc]; MIC; the minimal inhibitory concentration.) determined by the well diffusion assay. Values represent the mean ± standard deviation. Different letters mean significant differences between the NPEPp and the standard (*p* < 0.05).

## Discussion

A diabetic foot ulcer represents one of the most serious complications of diabetes mellitus; if not properly treated, it may progress to osteomyelitis, gangrene, and ultimately limb amputation, contributing to increased mortality rates^37^. The most frequently isolated bacterial strains include *Pseudomonas* spp. and *S. aureus*^38^, with *Pseudomonas* reported as the predominant gram-negative organism in diabetic foot infections across several Middle Eastern populations^39^. Although fungal infection is less common, *C. albicans* has been repeatedly identified among clinically relevant fungal isolates in diabetic foot infections^40^.

*C. japonica* is globally recognized for its antimicrobial and nutritional properties^12^. In this study, a metabolomic GC-MS-based approach was used to characterize non-polar metabolites in *C. japonica* fruit tissue (peel and pulp), revealing 63 saponifiable and 55 unsaponifiable compounds. Multivariate PCA analysis clearly distinguished between the two tissues, indicating distinct metabolic pathways and potential ecological roles.

Chemometric profiling further demonstrated tissue-specific enrichment of bioactive constituents. Alkyl benzenes were present in both tissues but were markedly higher in USFPp (58.53%), where PCA highlighted phenyl decane derivatives as key discriminants **(**Fig. 4. **B)**. These compounds have been previously associated with antimicrobial defense mechanisms in plants, supporting their potential role in pathogen suppression^41,42^. Among these alkyl benzenes are phenyl decane derivatives, which showed antifungal and antibacterial activities^43^.

Oxygenated monoterpenes, including limonene oxide, linalool, and others, were detected in both tissues, with slightly higher abundance in the peel (Fig. 4-B**).** These Oxygenated monoterpenes exhibited varying degrees of antibacterial activities^44^. Their antimicrobial relevance has been well documented, particularly enhanced activity against Gram-negative bacteria, which has been attributed to oxygenated functional groups that increase membrane permeability^45^.

More importantly, PCA-based GC-MS indicated that the pulp fraction (FAEPp) is characterized by higher levels of hexadecanoic acid methyl ester, trimethyl citrate, and dimethyl azelate (Fig. 5-B**).** These metabolites likely contribute collectively to the stronger antimicrobial activity observed in the pulp compared to the peel. Fatty acid methyl esters, such as hexadecanoic acid methyl ester, are known to disrupt microbial membranes^46–48^, an acknowledged mechanism established for long-chain unsaturated fatty acids. Additionally, citrate derivatives may enhance activity through metal ion chelation, thereby destabilizing cell envelope integrity and biofilm structure^49,50^. This may weaken bacterial defenses and increase susceptibility to other antimicrobial agents, particularly in Gram-negative strains^51^. Furthermore, azelaic acid derivatives further complement this effect by inhibiting key microbial enzymes involved in DNA and protein synthesis^52,53^. Approved by the FDA, azelaic acid derivatives are widely used in dermatological treatment, exhibiting in vitro bacteriostatic activity against several bacteria, including *P. aeruginosa*, *S. aureus*, *Staphylococcus epidermidis*, and *Propionibacterium acnes*^54^.

Although the observed MIC values obtained (0.938–3.75 mg/mL) indicate only moderate antimicrobial potency, they are consistent with previously reported values for other *Citrus* extracts, which typically range from 2.5 to 25 mg/mL against *S. aureus* and *P. aeruginosa*^51,52^. This moderate activity is expected, given the complex, slightly non-purified nature of the lipophilic extracts, where multiple compounds may act synergistically or competitively, influencing overall bioactivity.

Overall, the modest but broad-spectrum antimicrobial activity of the pulp fraction is likely driven by a synergistic interplay between membrane-disrupting active fatty acid esters, citrate with metal-chelating properties, and azelate acting as an enzyme inhibitor, rather than a single dominant constituent.

Finally, this study serves as a preliminary screening of tissue-specific bioactivity in *C. japonica*. Further fractionation and isolation of pure compounds are required to establish definitive structure–activity relationships and to validate the therapeutic potential of the most active metabolites.

## Conclusion

This study provides the first GC-MS-based metabolomic comparison of the non-polar fractions from the peel and pulp of *C. japonica*, supported by GNPS molecular networking and PCA chemometrics. The pulp fraction was enriched in bioactive metabolites, including trimethyl citrate, dimethyl azelate, and methyl palmitate, and exhibited weak to moderate in vitro antimicrobial activity against three pathogens commonly associated with diabetic foot infections (*P. aeruginosa*, *S. aureus*, and *C. albicans*). These findings suggest that the observed activity is driven by synergistic effects of multiple compounds affecting microbial membranes, metal ion balance, and enzymatic pathways. In conclusion, the pulp fraction of *C. japonica* represents a promising source of antimicrobial lead compounds, warranting further isolation, in vitro assays (biofilm, cytotoxicity, and wound healing), and in vivo validation.

## Supplementary Information

Below is the link to the electronic supplementary material.

Supplementary Material 1

## Data Availability

The datasets supporting the conclusions of this article are publicly available via the Global Natural Products Social Molecular Networking (GNPS) platform. The specific analysis job can be accessed at the following permanent link: for Unsaponifiable fractions (UCSD Computational Mass Spectrometry Website ) and for fatty acids fractions (UCSD Computational Mass Spectrometry Website).

## References

[1] McDermott, K., Fang, M., Boulton, A. J. M., Selvin, E. & Hicks, C. W. Etiology, Epidemiology, and Disparities in the Burden of Diabetic Foot Ulcers. *Diabetes Care*. **46**, 209–211 (2023). 10.2337/dci22-0043 Preprint at. 36548709 PMC9797649

[2] Surboyo, M. D. C. et al. Number of macrophages and transforming growth factor β expression in citrus limon L. Tlekung peel oil-treated traumatic ulcers in diabetic rats. *Trop. J. Pharm. Res.* **18**, 1427–1433 (2019).

[3] Raspo, M. A., Vignola, M. B., Andreatta, A. E. & Juliani, H. R. Antioxidant and antimicrobial activities of citrus essential oils from Argentina and the United States. *Food Biosci.* **36**, 100651 (2020).

[4] Agarwal, P. *et al. Citrus* Essential Oils in Aromatherapy: Therapeutic Effects and Mechanisms. *Antioxidants* 11 Preprint at (2022). 10.3390/antiox11122374 10.3390/antiox11122374 PMC9774566 36552586

[5] Ragheb, A. Y. et al. MS/MS-based molecular networking for mapping the chemical diversity of the pulp and peel extracts from *Citrus japonica* Thunb.; in vivo evaluation of their anti-inflammatory and anti-ulcer potential. *Scientific African* **20**, (2023).

[6] Wang, Y. W. et al. Chemical composition and antimicrobial activity of the essential oil of kumquat (*Fortunella crassifolia* swingle) peel. *Int. J. Mol. Sci.* **13**, 3382–3393 (2012). 22489157 10.3390/ijms13033382 PMC3317718

[7] Satyal, P., Paudel, P., Limbu, K. & Setzer, W. N. Leaf essential oil composition of *Citrus japonica* from Nepal. *J. Essent. Oil-Bearing Plants*. **15**, 357–359 (2012).

[8] Kassim, A., Workneh, T. S. & Laing, M. D. Development and Evaluation of a Small-scale In-field Integrated Postharvest Citrus Treatment Unit - Part 1. *International J. Food Engineering* **13**, (2017).

[9] Phala, K. et al. Inhibition of Kumquat Postharvest Fungi through Vapor Contact with Spearmint Essential Oil and Carvone. *ACS Agricultural Sci. Technol.* **2**, 330–339 (2022).

[10] Liu, X. et al. The accumulation and composition of essential oil in kumquat peel. *Sci. Hort.* **252**, 121–129 (2019).

[11] Lou, S. N. & Ho, C. T. Phenolic compounds and biological activities of small-size citrus: Kumquat and calamondin. *Journal of Food and Drug Analysis* vol. 25 162–175 Preprint at (2017). 10.1016/j.jfda.2016.10.024 10.1016/j.jfda.2016.10.024 PMC9333435 28911534

[12] Sumonrat, C., Suphitchaya, C. & Tipparat, H. Antimicrobial activities of essential oils and crude extracts from tropical Citrus spp. against food-related microorganisms. *Songklanakarin J. Sci. Technol.* **30**, 125–131 (2008).

[13] Jayaprakasha, G., Murthy, K. C., Demarais, R. & Patil, B. Inhibition of prostate cancer (LNCaP) cell proliferation by volatile components from Nagami Kumquats. *Planta Médica*. **78**, 974–980 (2012). 22673830 10.1055/s-0031-1298619

[14] Schirra, M. et al. Influence of postharvest hot water treatment on nutritional and functional properties of Kumquat (*Fortunella japonica* Lour. swingle cv. ovale) fruit. *J. Agric. Food Chem.* **56**, 455–460 (2008). 18163539 10.1021/jf0714160

[15] Al-Rubaye, A. F., Hameed, I. H., Kadhim, M. J. A. & Review Uses of Gas Chromatography-Mass Spectrometry (GC-MS) Technique for Analysis of Bioactive Natural Compounds of Some Plants. *Int. J. Toxicol. Pharmacol. Res.* **9**, 81–85 (2017).

[16] Ragab, N. A. et al. Chemical characterization of *Melilotus messanensis* (L.) all.: Antioxidant, antidiabetic and antimutagenic effects in alloxan induced diabetic rats. *Biocatal. Agric. Biotechnol.* **33**, 101976 (2021).

[17] Waly, D. A. et al. Avocado fruit peel and flesh as antioxidant and anti-inflammatory agents: A comparative phytochemical and in vitro study. *South. Afr. J. Bot.* **179**, 334–344 (2025).

[18] Adams, R. P. *Identification of Essential Oil Components by Gas Chromatography/Quadruple Mass Spectroscopy*. (Allured Publishing Corporation, USA).

[19] Liu, J., Clarke, J. A., McCann, S., Hillier, N. K. & Tahlan, K. Analysis of Streptomyces Volatilomes Using Global Molecular Networking Reveals the Presence of Metabolites with Diverse Biological Activities. *Microbiology Spectrum* **10**, (2022). 10.1128/spectrum.00552-22 PMC9431705 35900081

[20] Aksenov, A. A. et al. Auto-deconvolution and molecular networking of gas chromatography–mass spectrometry data. *Nat. Biotechnol.* **39**, 169–173 (2021). 33169034 10.1038/s41587-020-0700-3 PMC7971188

[21] El-Sawi, S. A. et al. Principal component analysis of phenolic compounds of grape waste parts and correlations to their bioassays. *Biocatal. Agric. Biotechnol.* **51**, 102780 (2023).

[22] Hafez, A. I., Ali, H. M., Sabry, R. M. & El-Masry, H. M. Abd El-Gawad, W. M. Generation of novel, hygienic, inhibitive, and cost-effective nanostructured Core-shell pigments. *Prog. Org. Coat.* **175**, 107325 (2023).

[23] El-Masry, H. M. et al. Phenazine-Producing *Pseudomonas aeruginosa* OQ158909: A Promising Candidate for Biological Activity and Therapeutic Applications. *Egypt. J. Chem.* **66**, 281–303 (2023).

[24] McFarland, J. The nephelometer: an instrument for estimating the number of bacteria in suspensions used for calculating the opsonic index and for vaccines. *J. Am. Med. Assoc.* **49**, 1176–1178 (1907).

[25] Hamoda, D. M. et al. Plasma Technique Application for Coating Non - Woven Fabric (SMS) by (CaSiO3/CuO) Nano Particles for Biomedical Sector. *Egypt. J. Chem.* **0**, 0–0 (2022).

[26] El-Anssary, A. A., Raoof, G. F. A., Saleh, D. O. & El-Masry, H. M. Bioactivities, physicochemical parameters and GC/MS profiling of the fixed oil of *Cucumis melo* L seeds: A focus on anti-inflammatory, immunomodulatory, and antimicrobial activities. *J. HerbMed Pharmacol.* **10**, 476–485 (2021).

[27] Tohamy, H. A. S. & El-Masry, H. M. Fluffy-like amphiphilic graphene oxide and its effects on improving the antibacterial activity and thermal outstanding of ethyl cellulose /polyvinyl alcohol hydrogel film. *BMC Chem.* **18**, 116 (2024). 38926782 10.1186/s13065-024-01221-3 PMC11210031

[28] Saquib, S. A. et al. Evaluation and comparison of antibacterial efficacy of herbal extracts in combination with antibiotics on periodontal pathobionts: An in vitro microbiological study. *Antibiotics* **8**, (2019). 10.3390/antibiotics8030089 PMC6783985 31266146

[29] Ledesma, R., Valero-Mora, P. & Macbeth, G. The Scree Test and the Number of Factors: a Dynamic Graphics Approach. *The Span. J. Psychology* **18**, (2015). 10.1017/sjp.2015.13 26055575

[30] Sébastien Baud, L. L. Physiological and developmental regulation of seed oil production. *Prog. Lipid Res.* **49**, 235–249 (2010). 20102727 10.1016/j.plipres.2010.01.001

[31] Sweetlove, L. J., Beard, K. F. M., Nunes-Nesi, A., Fernie, A. R. & Ratcliffe, R. G. Not just a circle: flux modes in the plant TCA cycle. *Trends Plant. Sci.* **15**, 462–470 (2010). 20554469 10.1016/j.tplants.2010.05.006

[32] Kunst, L. & Samuels, A. L. Biosynthesis and secretion of plant cuticular wax. *Prog. Lipid Res.* **42**, 51–80 (2003). 12467640 10.1016/s0163-7827(02)00045-0

[33] Soleimani, H., Mostowfizadeh-Ghalamfarsa, R., Ghanadian, M., Karami, A. & Cacciola, S. O. Defense Mechanisms Induced by Celery Seed Essential Oil against Powdery Mildew Incited by *Podosphaera fusca* in Cucumber. *J. Fungi*. **10**, 17 (2024). 10.3390/jof10010017 PMC10817264 38248927

[34] Turlings, T. C. J. & Erb, M. Tritrophic Interactions Mediated by Herbivore-Induced Plant Volatiles: Mechanisms, Ecological Relevance, and Application Potential. *Ann. Rev. Entomol.* **17**, 14 (2025). 10.1146/annurev-ento-020117-043507 29324043

[35] Borges, R. M., Bessière, J. M. & Ranganathan, Y. Diel Variation in Fig Volatiles Across Syconium Development: Making Sense of Scents. *J. Chem. Ecol.* **39**, 630–642 (2013). 23609162 10.1007/s10886-013-0280-5

[36] Sweetlove, L. J. & Fernie, A. R. The Spatial Organization of Metabolism Within the Plant Cell. *Annu. Rev. Plant Biol.* **64**, 723–746 (2013). 23330793 10.1146/annurev-arplant-050312-120233

[37] Shaheen, M. M. A., Dahab, A., Abu Fada, S., Idieis, R. & M. & Isolation and characterization of bacteria from diabetic foot ulcer: amputation, antibiotic resistance and mortality rate. *Int. J. Diabetes Developing Ctries.* **42**, 529–537 (2022). 10.1007/s13410-021-00997-7 PMC8431256 34522073

[38] Kaimkhani, G. M. et al. Pattern of Infecting Microorganisms and Their Susceptibility to Antimicrobial Drugs in Patients with Diabetic Foot Infections in a Tertiary Care Hospital in Karachi, Pakistan. *Cureus* **10**, 6 (2018). 10.7759/cureus.2872 PMC6110415 30155375

[39] Obeid, M. et al. Epidemiology and susceptibility profiles of diabetic foot infections in five hospitals in Lebanon. *J. Infect. Developing Ctries.* **12**, 347–351 (2018). 10.3855/jidc.10063 31865297

[40] Missoni, E. M. et al. Fungal infection in diabetic foot ulcers [4]. *Diabet. Med.* **22**, 1124–1125 (2005). 16026387 10.1111/j.1464-5491.2005.01611.x

[41] Benítez, E. et al. Bottom-up effects on herbivore-induced plant defences: A case study based on compositional patterns of rhizosphere microbial communities. *Scientific Reports* **7**, (2017). 10.1038/s41598-017-06714-x PMC5524984 28740172

[42] EL-Hefny, M., Mohamed, A. A., Salem, M. Z. M., El-Kareem, A., Ali, H. M. & M. S. M. & Chemical composition, antioxidant capacity and antibacterial activity against some potato bacterial pathogens of fruit extracts from *Phytolacca dioica* and *Ziziphus spina-christi* grown in Egypt. *Sci. Hort.* **233**, 225–232 (2018).

[43] Asghari, G., Jalali, M. & Sadoughi, E. *Antimicrobial Activities and Chemical Composition of Essential Oil from the Seeds of Artemisia aucheri* Boiss. *Jundishapur J. Nat. Pharm. Prod.* **7**, 11 (2012). 24624145 PMC3941861

[44] Kotan, R., Kordali, S. & Cakir, A. Screening of Antibacterial Activities of Twenty-One Oxygenated Monoterpenes. *Zeitschrift für Naturforschung C*. **62**, 507 (2007). 10.1515/znc-2007-7-808 17913064

[45] Guimarães, A. C. et al. Antibacterial activity of terpenes and terpenoids present in essential oils. *Molecules* **24**, 2471 (2019). 31284397 10.3390/molecules24132471 PMC6651100

[46] Davoodbasha, M. A., Edachery, B., Nooruddin, T., Lee, S. Y. & Kim, J. W. An evidence of C16 fatty acid methyl esters extracted from microalga for effective antimicrobial and antioxidant property. *Microb. Pathog.* **115**, 233–238 (2018). 29277474 10.1016/j.micpath.2017.12.049

[47] Desbois, A. P. & Smith, V. J. Antibacterial free fatty acids: Activities, mechanisms of action and biotechnological potential. *Appl. Microbiol. Biotechnol.* **85**, 1629–1642 (2010). 19956944 10.1007/s00253-009-2355-3

[48] Shaaban, M. T., Ghaly, M. F. & Fahmi, S. M. Antibacterial activities of hexadecanoic acid methyl ester and green-synthesized silver nanoparticles against multidrug-resistant bacteria. *J. Basic Microbiol.* **61**, 557–568 (2021). 33871873 10.1002/jobm.202100061

[49] Nagaoka, S., Murata, S., Kimura, K., Mori, T. & Hojo, K. Antimicrobial activity of sodium citrate against Streptococcus pneumoniae and several oral bacteria. *Lett. Appl. Microbiol.* **51**, 546–551 (2010). 20849395 10.1111/j.1472-765X.2010.02932.x

[50] Lieleg, O., Caldara, M., Baumgärtel, R. & Ribbeck, K. Mechanical robustness of *Pseudomonas aeruginosa* biofilms. *Soft Matter*. **7**, 3307–3314 (2011). 21760831 10.1039/c0sm01467b PMC3134232

[51] Su, L. C., Xie, Z., Zhang, Y., Nguyen, K. T. & Yang, J. Study on the antimicrobial properties of citrate-based biodegradable polymers. *Front. Bioeng. Biotechnol.* **2**, 23 (2014). 25023605 10.3389/fbioe.2014.00023 PMC4090902

[52] Petrovici, A. G. et al. A Comprehensive Review of Azelaic Acid Pharmacological Properties, Clinical Applications, and Innovative Topical Formulations. *Pharmaceuticals* **18**, 1273 (2025). 41011144 10.3390/ph18091273 PMC12472904

[53] Sauer, N. et al. The multiple uses of azelaic acid in dermatology: mechanism of action, preparations, and potential therapeutic applications. *Adv. Dermatol. Allergol*. **40**, 716–724 (2023). 10.5114/ada.2023.133955 PMC10809820 38282869

[54] Feng, X. et al. Azelaic Acid: Mechanisms of Action and Clinical Applications. *Clin. Cosmet. Invest. Dermatology*. **17**, 2359–2371 (2024). 10.2147/CCID.S485237 PMC11512533 39464747

