# Supplementary material for: Molecular networking-based GC/MS profiling of Citrus japonica Thunb. peel and pulp lipophilic fractions and their antimicrobial potential against diabetic foot ulcer pathogens
Source: Sci Rep. 2026 Jun 4;16:17328. doi: 10.1038/s41598-026-55298-y (PMC13237011; doi:10.1038/s41598-026-55298-y)
Supplement: Supplementary file 1 — Supplementary Material 1 [file 41598_2026_55298_MOESM1_ESM.docx]

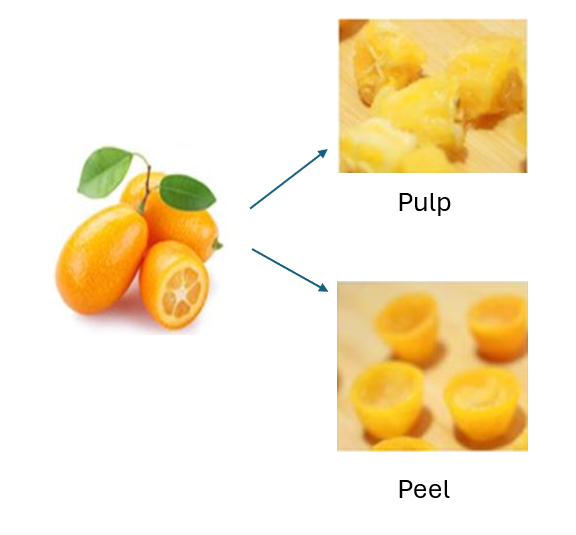


Fig.(S1): Photograph of the whole fruit, peel and pulp


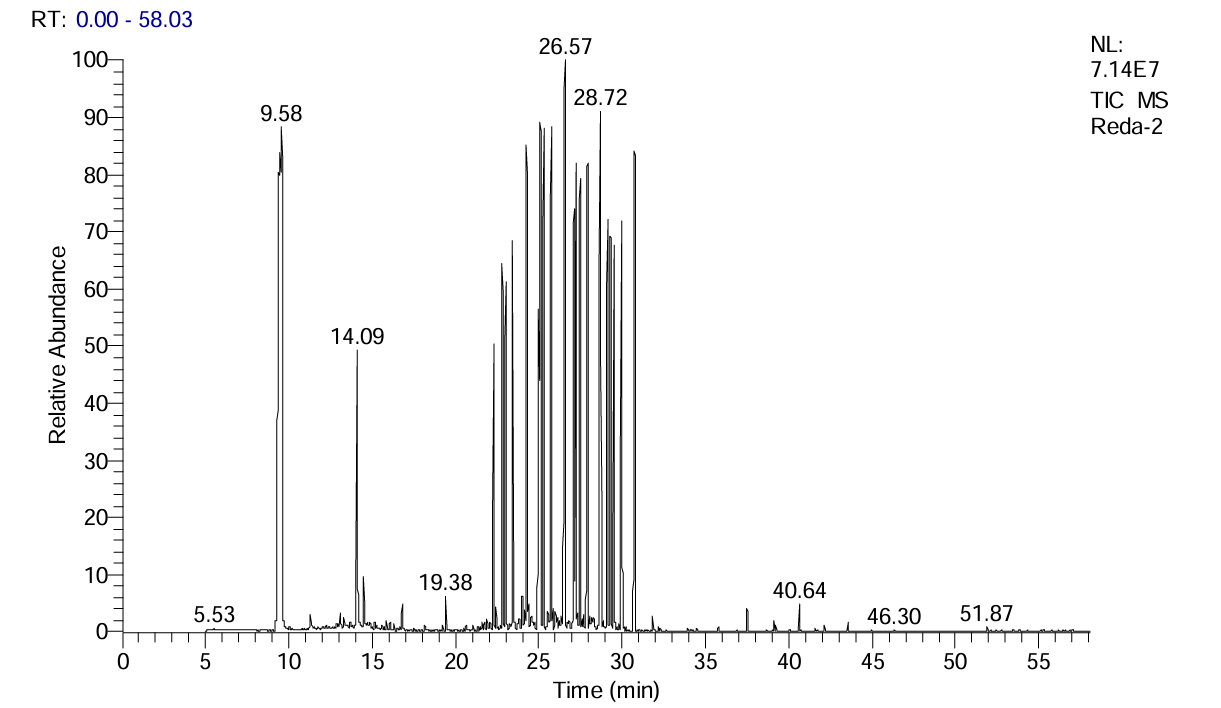


Fig.(S2): Total ion chromatogram of GC-MS analyses of the unsaponifiable matter of pulp


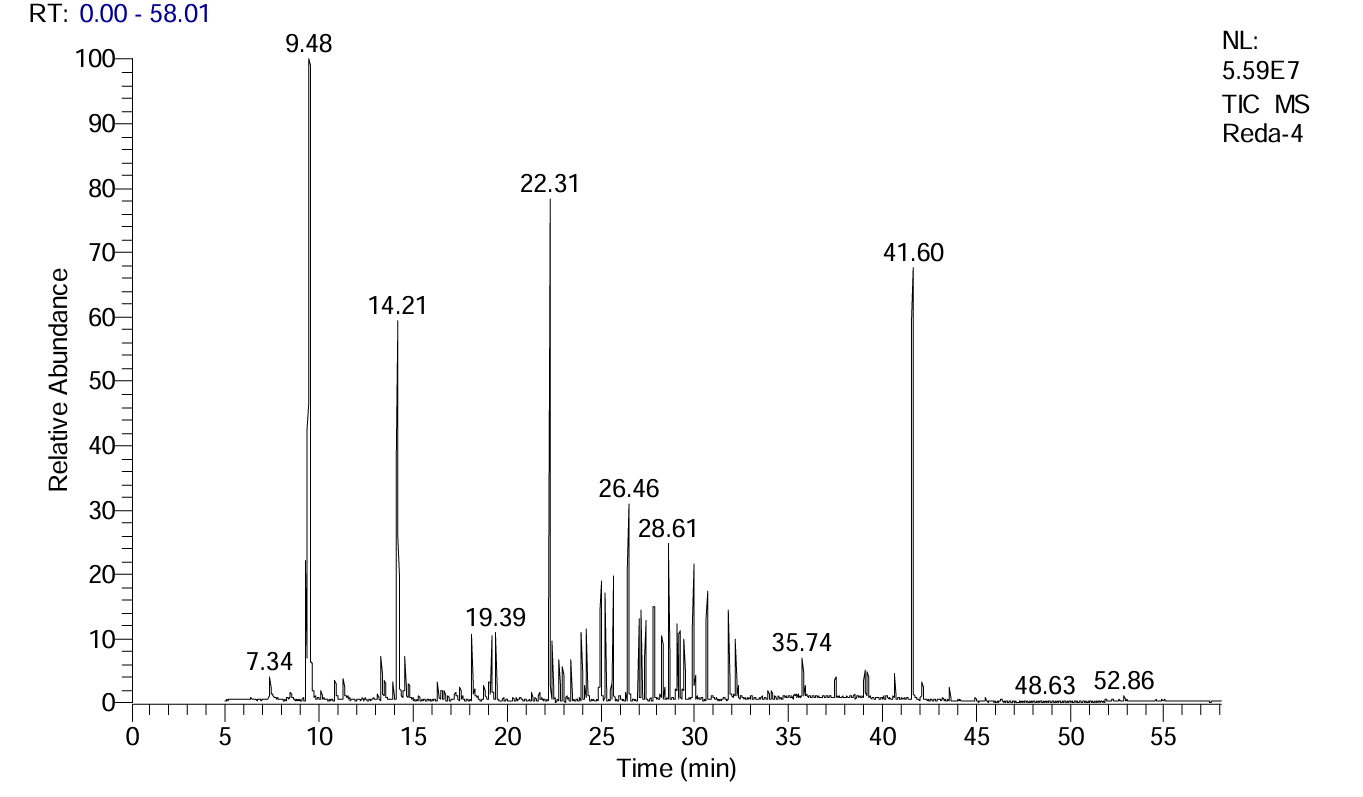


Fig.(S3): Total ion chromatogram of GC-MS analyses of the unsaponifiable matter of peel


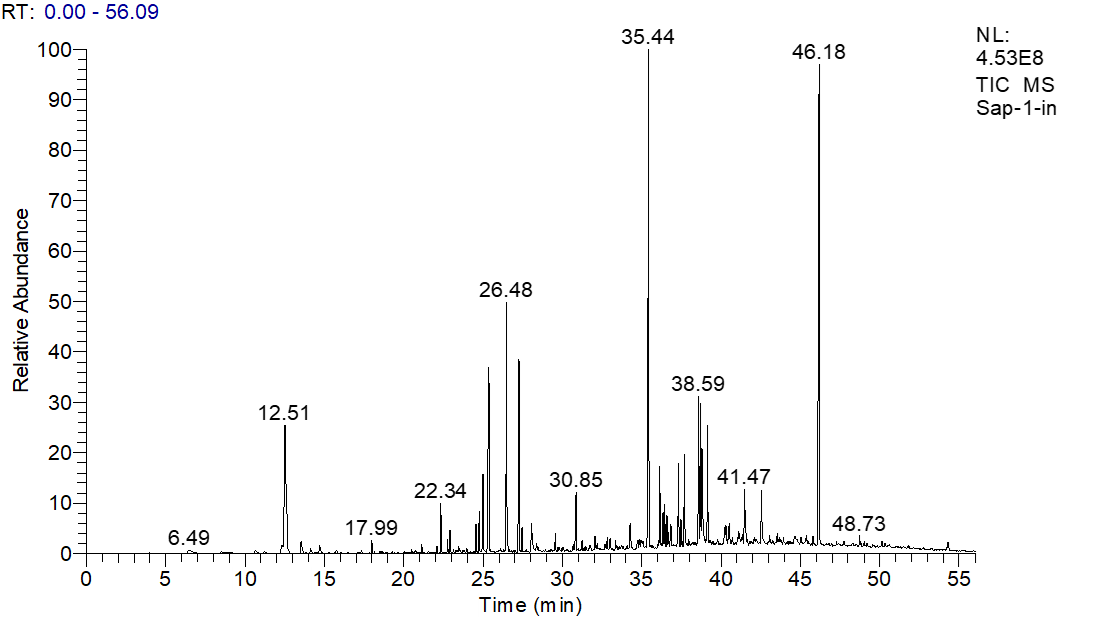


Fig.(S4): Total ion chromatogram of GC-MS analyses of the fatty acids of pulp

Fig.(S5): Total ion chromatogram of GC-MS analyses of the fatty acids of peel

**Saponified supplementary materials**


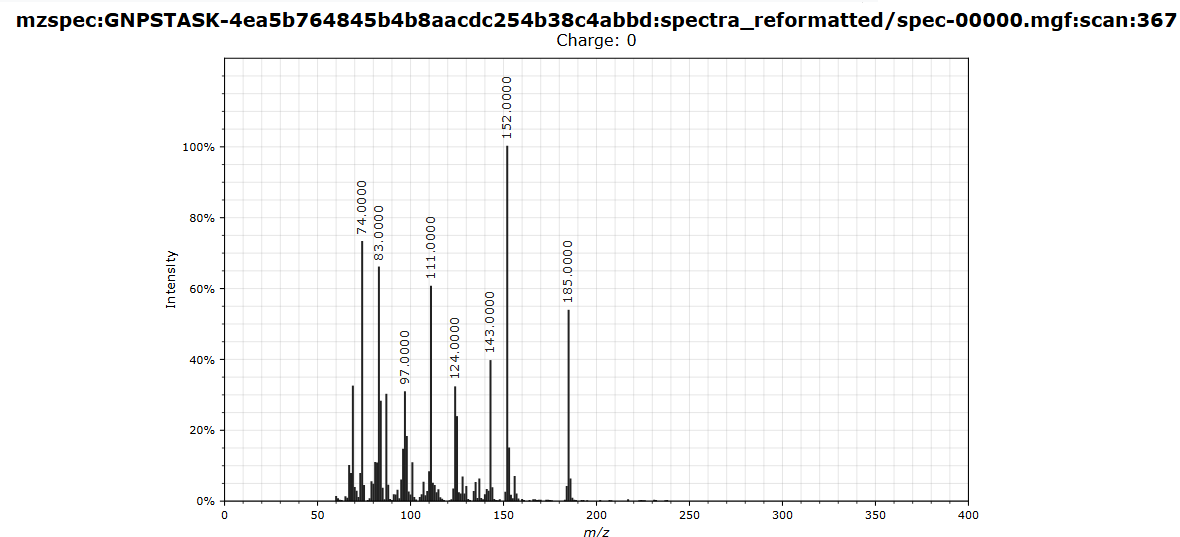


Fig.(S6): Mass spectrum of Nonanedioic acid, dimethyl ester (Dimethyl azelate)


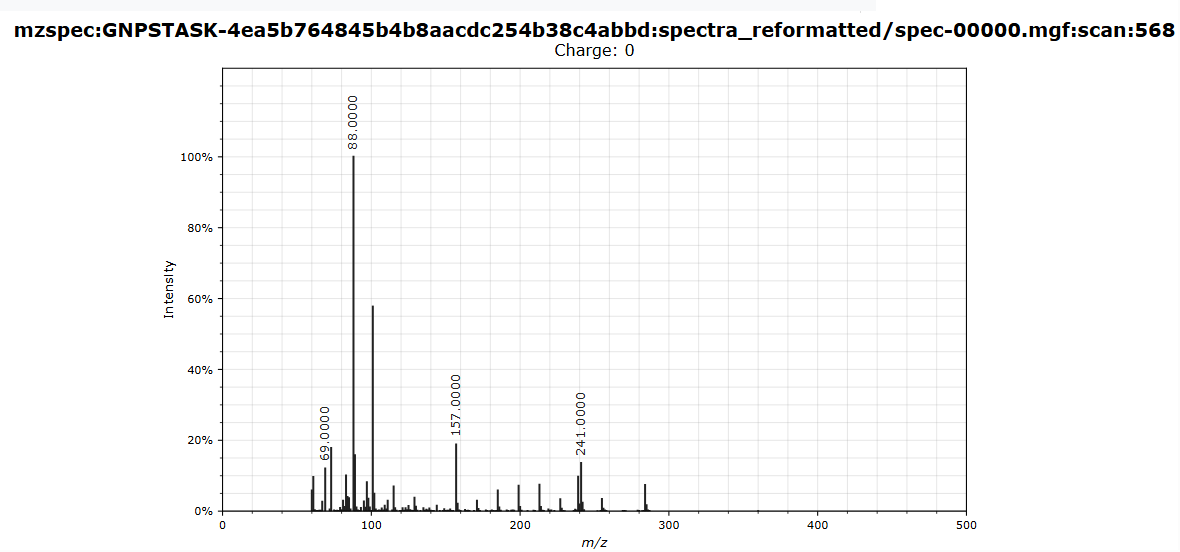
 Fig.(S7): Mass spectrum of Hexadecanoic acid, ethyl ester


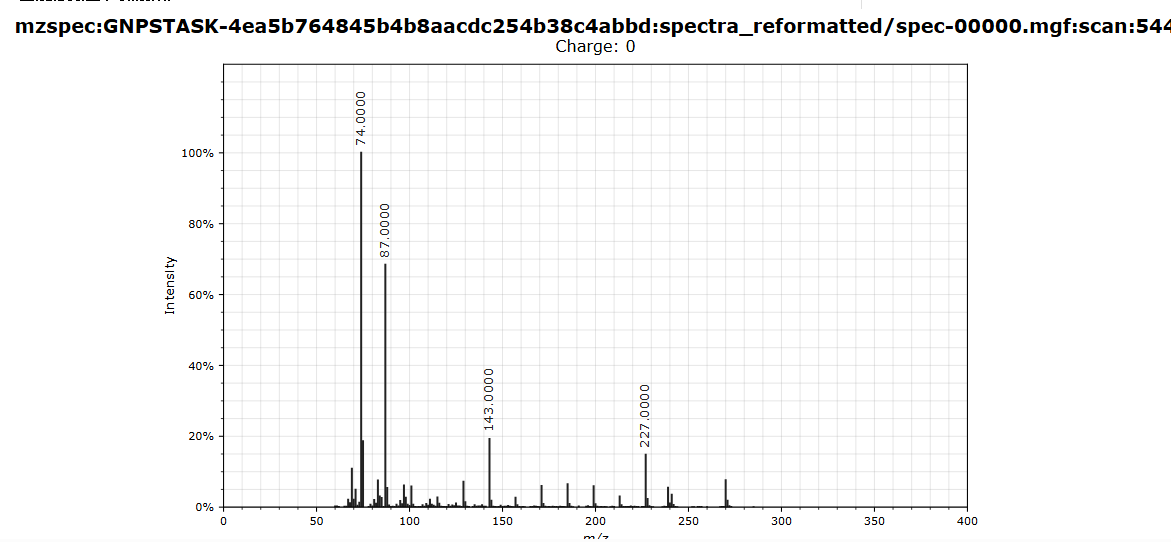
 Fig.(S8): Mass spectrum of Hexadecenoic acid methyl ester


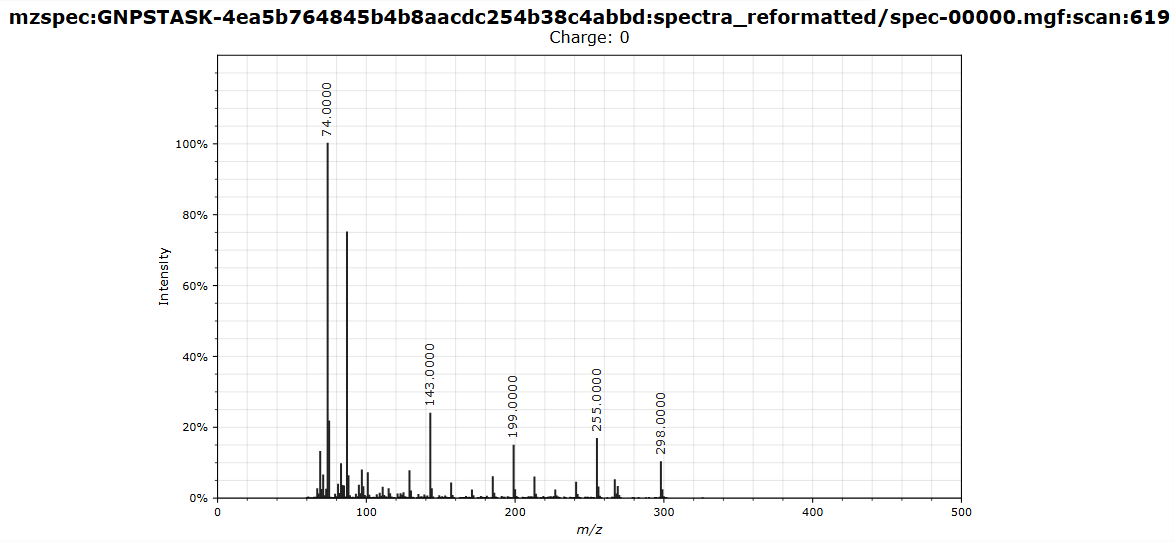
 Fig.(S9): Mass spectrum of Octadecanoic acid methyl ester (Methyl Stearate)


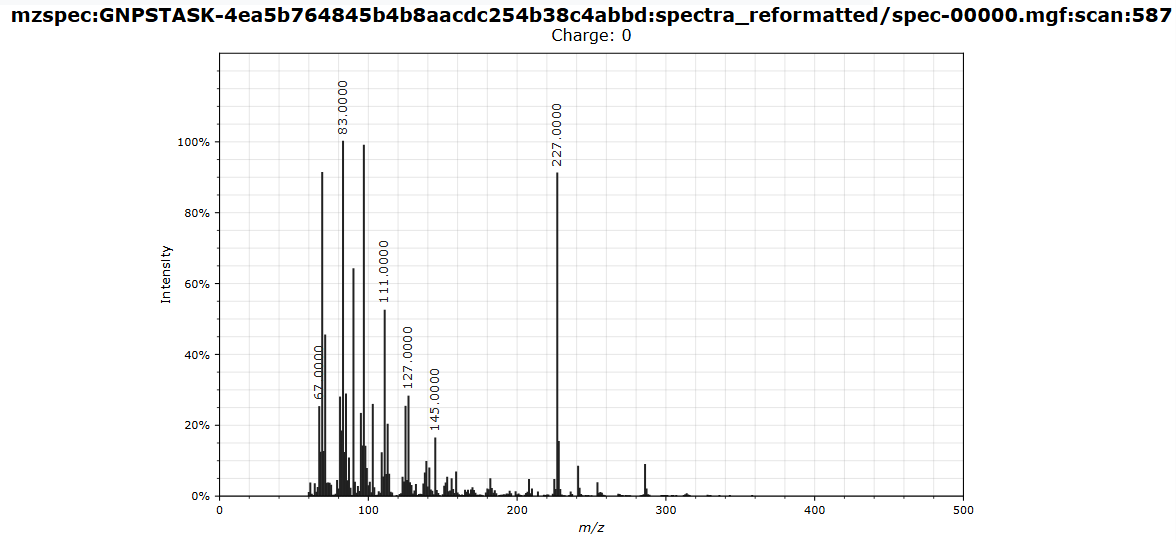
 Fig.(S10): Mass spectrum of Methyl 2-Hydroxyhexadecanoate


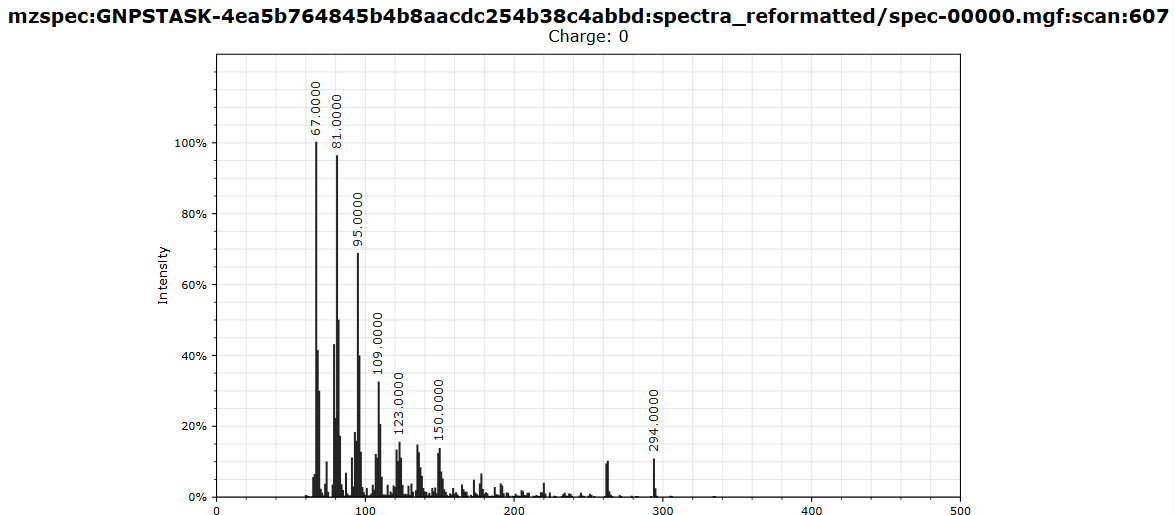


Fig.(S11): Mass spectrum of 9,12-Octadecadienoic acid, methyl ester


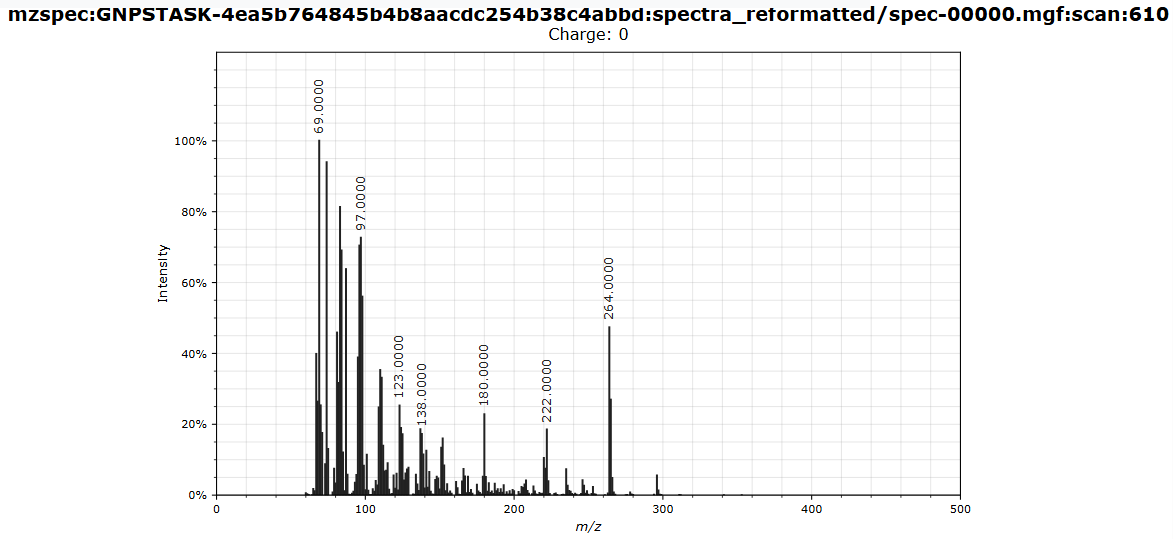
 Fig.(S12): Mass spectrum of 9-Octadecenoic acid, methyl ester.

**unsaponified fractions. supplementary materials**


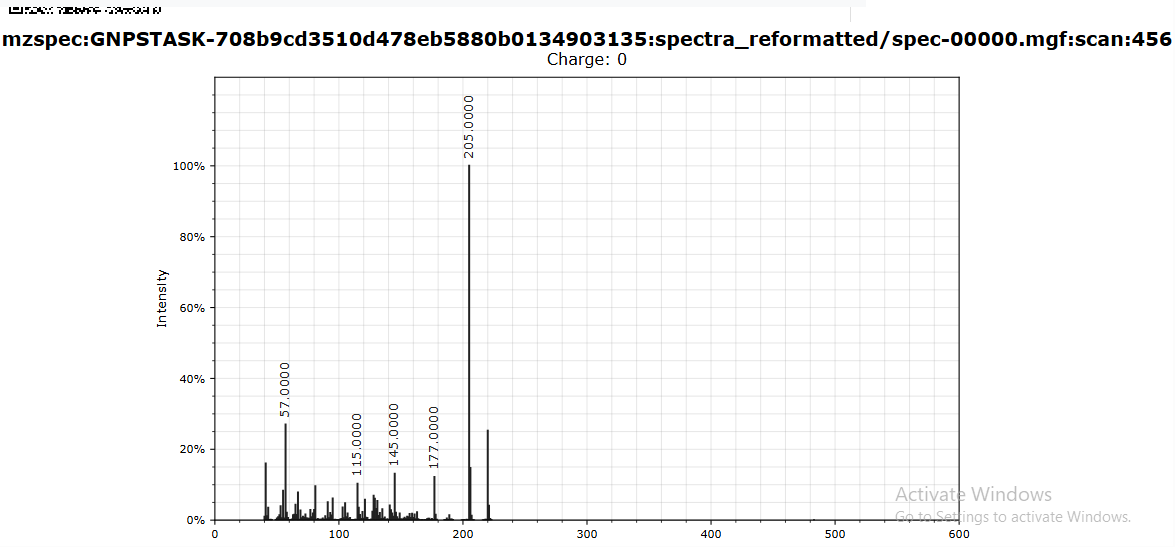
 Fig.(S13): Mass spectrum of Butylated hydroxy toluene


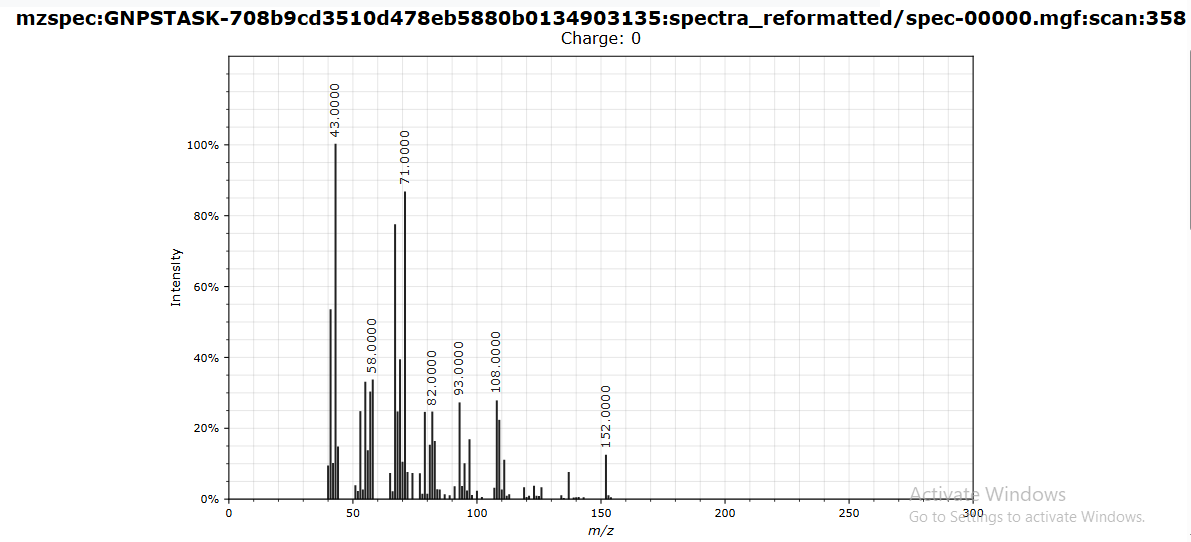


Fig.(S14): Mass spectrum of Limonene-1,2-Oxide


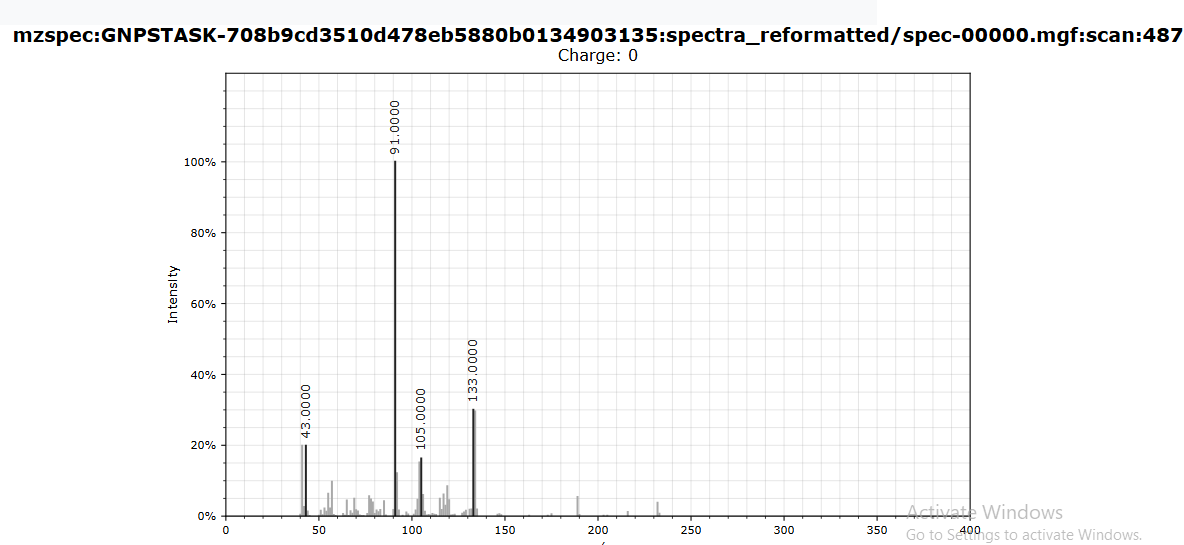
 Fig.(S15): Mass spectrum of 1-Pheny undecane


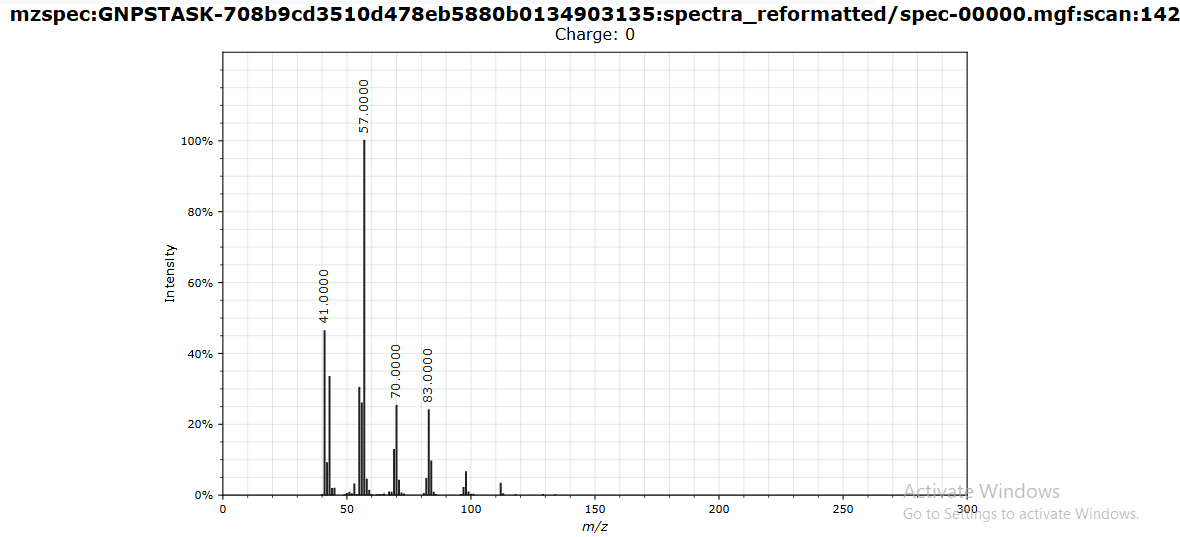
 Fig.(S16): Mass spectrum of 2-Ethyl -1-Hexanol


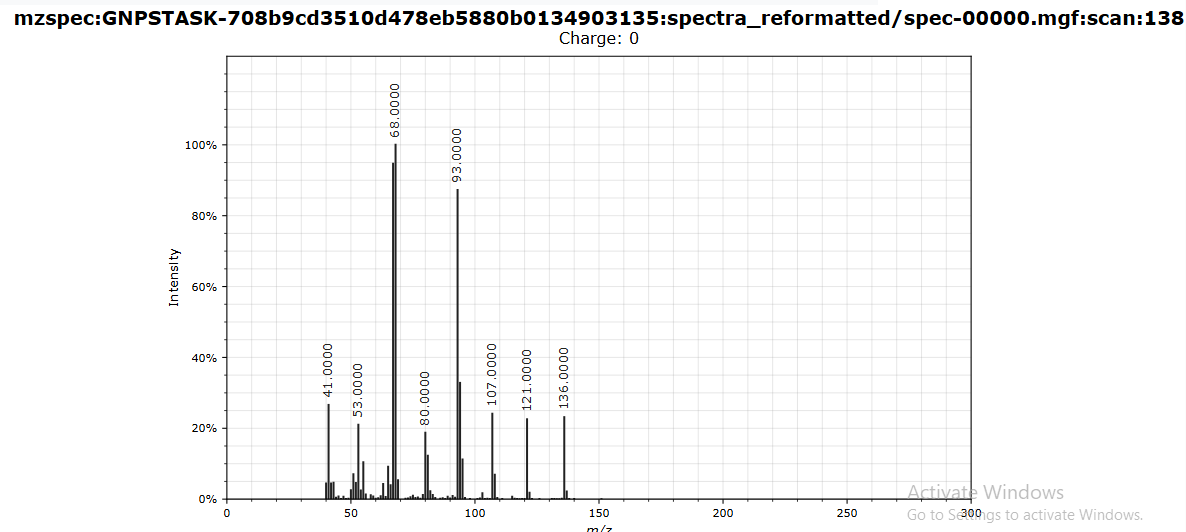
 Fig.(S17): Mass spectrum of Limonene


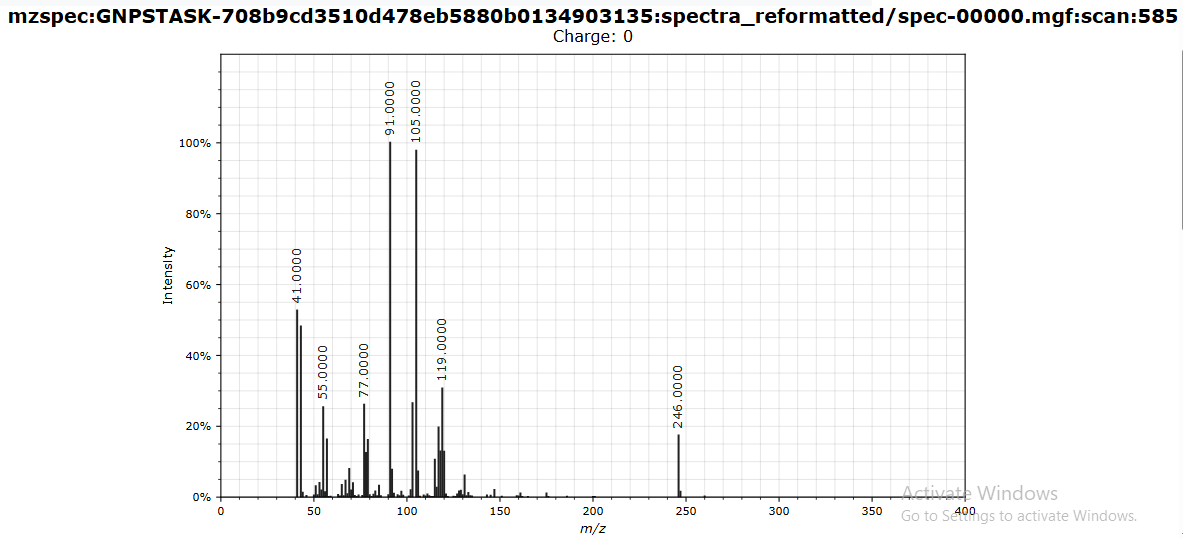


Fig.(S18): Mass spectrum of 2-Phenyldodecane


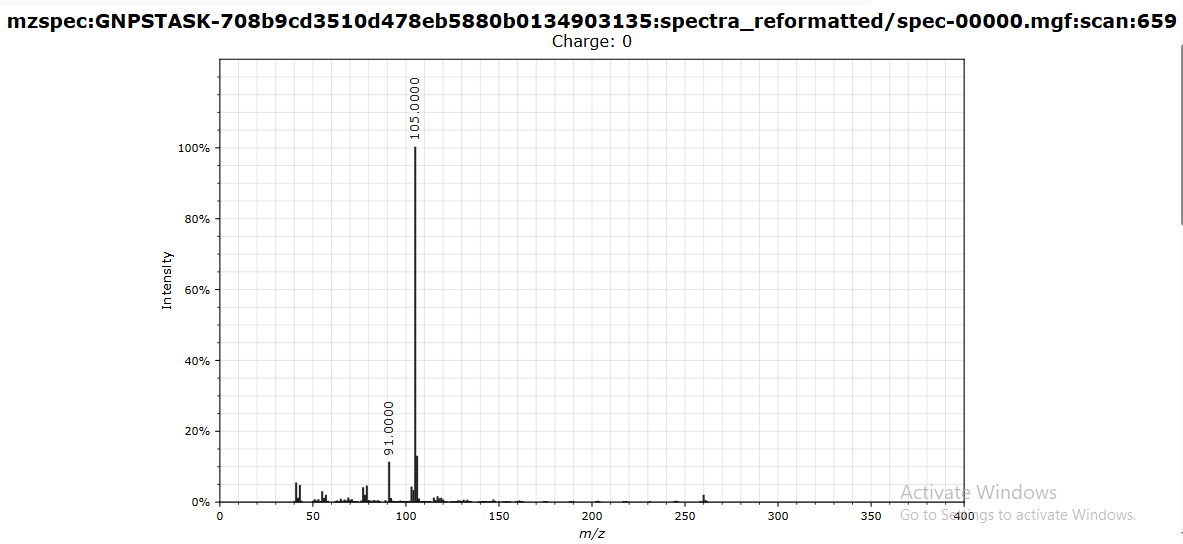


Fig.(S19): Mass spectrum of 2-Phenyl tridecane
